# Silent zero TE MR neuroimaging: Current state-of-the-art and future directions

**DOI:** 10.1016/j.pnmrs.2021.03.002

**Published:** 2021-03-26

**Authors:** Emil Ljungberg, Nikou L. Damestani, Tobias C. Wood, David J. Lythgoe, Fernando Zelaya, Steven C.R. Williams, Ana Beatriz Solana, Gareth J. Barker, Florian Wiesinger

**Affiliations:** a Department of Neuroimaging, Institute of Psychiatry, Psychology & Neuroscience, King’s College London, London, United Kingdom; b ASL Europe, GE Healthcare, Munich, Germany

**Keywords:** Zero Echo Time (ZTE), Silent MRI, Neuroimaging

## Abstract

Magnetic Resonance Imaging (MRI) scanners produce loud acoustic noise originating from vibrational Lorentz forces induced by rapidly changing currents in the magnetic field gradient coils. Using zero echo time (ZTE) MRI pulse sequences, gradient switching can be reduced to a minimum, which enables near silent operation. Besides silent MRI, ZTE offers further interesting characteristics, including a nominal echo time of TE = 0 (thus capturing short-lived signals from MR tissues which are otherwise MR-invisible), 3D radial sampling (providing motion robustness), and ultra-short repetition times (providing fast and efficient scanning). In this work we describe the main concepts behind ZTE imaging with a focus on conceptual understanding of the imaging sequences, relevant acquisition parameters, commonly observed image artefacts, and image contrasts. We will further describe a range of methods for anatomical and functional neuroimaging, together with recommendations for successful implementation.

## 1 Introduction

Magnetic Resonance Imaging (MRI) scanners produce loud acoustic noise because of Lorentz forces caused by rapidly changing currents in the magnetic field gradient coils used primarily for spatial localization [1]. The acoustic noise produced with conventional sequences is typically around 90–110 dBA (where dBA is dB on the A-weighted scale, which accounts for the sensitivity of the human ear at different frequencies) [2–4]. For fast imaging sequences, such as Echo Planar Imaging (EPI), it can even reach levels up to 130 dBA [5]. Since the Lorentz force scales with the main magnetic field strength, acoustic noise also increases at higher field strength [6].

It is generally acknowledged that the acoustic noise produced by the MRI scanner is one of the most unpleasant aspects of the scan experience for patients [7–12]. Given appropriate and correctly worn hearing protection [13,14] there are, to our knowledge, no studies showing permanent hearing loss after a single MRI scan [15,16]. However, studies have reported temporary effects on hearing following scans at both 1.5T [17] and 3T [3].

Exposure to the loud noise inside the MRI scanner for long periods of time is problematic for certain groups of individuals. For instance, to avoid motion artefacts in neonatal and paediatric MRI, scanning is preferably performed under natural sleep [18,19], and this requires reduction of the acoustic noise. For individuals with hyperacusis [20], i.e., perception of ordinary sounds as abnormally loud, the noise from the MRI scanning can cause discomfort. Studies have found hyperacusis to be prevalent in numerous conditions including tinnitus, migraines, and autism spectrum disorder [20–22].

In functional MRI (fMRI) studies, the acoustic noise is an additional confounding sensory stimulus, and can impact the blood-oxygen level dependent (BOLD) response as a function of both its loudness [23] and duration [24]. The effect of acoustic noise on the BOLD activation appears to vary based on the task performed [25–27], and resting state network identification can be impacted by the sparse-sampling technique employed [28,29]. There are also patient groups with auditory symptoms who are difficult to study using conventional fMRI, since the information related to the auditory stimulus of the study has to be extracted from the background noise, as has been described in studies of tinnitus [30] and Williams syndrome [31].

The acoustic noise from the MRI scanner can be reduced through hardware modifications, such as novel gradient designs and shielding [32–34]. Most MRI manufacturers also support a “quiet scanning” mode, typically using standard pulse sequences but with derated gradient performance for smoother temporal changes [35,36]. Despite all these improvements, the vast majority of MRI scans performed today still remain very loud and require the patient to wear hearing protection for additional noise suppression, with typical earplugs reducing the noise by 20–30 dBA [13].

**Fig. 1:**
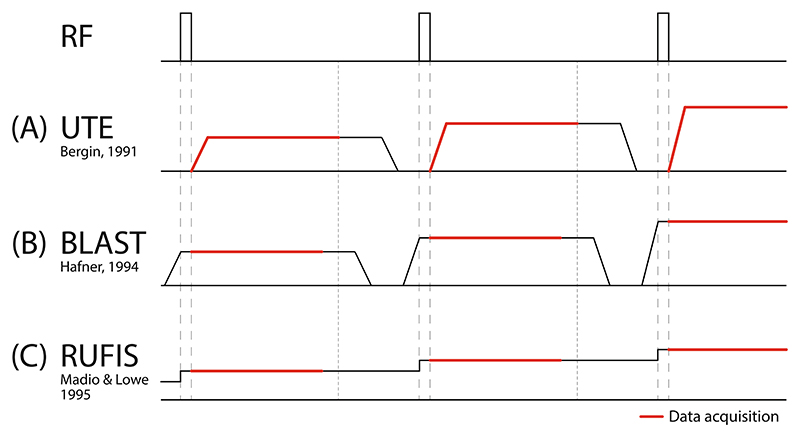
Simplified pulse sequences diagrams showing RF excitation and gradients in one dimension for (**A**) UTE, (**B**) BLAST and (**C**) RUFIS. In UTE imaging, RF excitation is performed prior to the readout gradients. In the BLAST pulse sequence, gradients are ramped up before RF excitation and ramped down after readout. In RUFIS, RF excitation is performed with the gradient on, as in BLAST, but without returning gradients to zero between excitations, hence minimizing gradient switching and allowing silent imaging.

Instead of lowering the acoustic noise of loud MRI sequences, it is also possible to diminish the generation of acoustic noise in the first place by minimising gradient switching, which can be achieved with Zero Echo Time (ZTE) pulse sequences [37,38]. The incremental development leading to silent ZTE imaging is best appreciated through comparison to the ultra-short echo time (UTE) pulse sequence [39,40], shown in Fig. 1. Both UTE and ZTE sequences are variants of a pulse-acquire free induction decay (FID) pulse sequence which do not create echo signals, and thus the term echo time (TE) can be confusing. When used for imaging, however, the delay between the middle of the RF pulse and the time at which the central k-space point (*k*_*0*_) is acquired determines the degree of T_2_* weighting, directly analogous to the TE in a gradient echo, and therefore the term TE will still be used.

In a UTE pulse sequence as shown in Fig. 1A, RF excitation is performed before gradients are ramped up and data acquisition begins after a short, non-negligible, delay time. Thus, *k*_*0*_ is acquired at a non-zero TE. The rapid gradient switching and simultaneous readout make UTE acoustically loud and susceptible to gradient delay and eddy current artefacts [41–43].

In ZTE imaging, first demonstrated with the Back-projection Low Angle Shot (BLAST) sequence [44] shown in Fig. 1B, the RF pulse is shifted to be applied after the gradient has reached the target amplitude, which means that *k*_*0*_ effectively is acquired at TE = 0. Hardware constraints, described in Section 2.1, makes it difficult to actually acquire this point, but the samples that are acquired are consistent with TE = 0 and thus the term ZTE is appropriate. By acquiring the FID during a constant gradient, ZTE imaging avoids the issues with the ramp-sampling characteristic in UTE imaging. However, similar to UTE imaging, BLAST will produce high acoustic noise due to the rapid gradient switching required for efficient imaging.

The ZTE Rotating Ultra-Fast Imaging Sequence (RUFIS) [45], shown in Fig. 1C, forms the basis for silent imaging with ZTE. It is similar to BLAST in that RF excitation is performed with the gradient on, but between excitations the readout gradient is ramped straight to the value required for the next set of sample points, for a faster acquisition and reduced gradient switching. The motivation of the original RUFIS method was ultra-fast imaging without extreme demands on the gradient system, but no specific emphasis was given to its potential for silent imaging.

An alternative to the hard pulse ZTE sequence shown in Fig. 1 is SWIFT (SWeep Imaging with Fourier Transformation) which uses swept RF pulse excitation [46,47]. SWIFT has been used less frequently than hard pulse ZTE for human neuroimaging applications primarily because of very demanding RF transmit-receive switching requirements. It has also been shown by Weiger et al. that while sweep excitation can produce higher flip angles, the SNR efficiency is actually equivalent to hard pulse excitation [47].

The purpose of this review is to give a comprehensive description of silent neuroimaging using ZTE MR pulse sequences. In the first half, the concept of ZTE will be reviewed from an MR physics point of view, together with descriptions of variations and extensions of the native ZTE pulse sequence. In the second half, practical neuroimaging applications of ZTE will be reviewed. We conclude with an outlook on the future of silent ZTE and potential translation to the clinic.

## 2 MRI with ZTE

This section describes the mechanics of the ZTE pulse sequence in terms of RF excitation and the k-space encoding. It lists common ZTE image artefacts and describes the acoustic noise behaviour of ZTE.

### 2.1 RF excitation in the presence of gradients

As described in Fig. 1, ZTE imaging requires RF excitation in the presence of the readout gradients, which leads to two unique challenges. Firstly, the excitation bandwidth of the RF pulse has to encompass the full receive imaging bandwidth to ensure uniform excitation independent of the readout direction [48]. Secondly, the finite time it takes to change from RF transmit to receive mode, typically referred to as the dead-time gap, causes some samples to be missed at the beginning of the readout [49].

A block RF pulse (i.e., rectangular) with pulse width *τ*_*TX*_ produces a sinc-shaped excitation profile along the readout direction according to

(1) $$P(\bar{r})=\alpha \cdot \text{sinc}\left(\gamma \cdot \tau_{TX}\cdot \bar{G}\cdot \bar{r}\right)\;\text{with}\;\text{sinc}(x)=\frac{\sin(\pi x)}{\pi x}$$

where *α* = *γB*_1_*τ*_*TX*_ is the nominal flip angle, *γ* is the gyromagnetic ratio, *B*_1_ is the RF excitation amplitude, $\bar{G}$ is the spoke gradient vector, and $\bar{r}$ is the position vector [48]. The excitation profile rotates in synchrony with the readout spoke direction and the effective flip angle progressively deviates from the nominal flip angle (*α*) with increasing distance from the isocentre. In order to avoid the first zero crossing of the sinc-shaped excitation profile falling within the field of view (FOV) (i.e., $\gamma \cdot \tau_{TX}\cdot |\bar{G}|\cdot FOV/2<1$), the excitation pulse width needs to be shorter than twice the dwell time (*dw*)

(2) $$\tau_{TX}<2\cdot dw,\;\text{with}\;dw=\frac{1}{BW_{RX}},\;\text{and}\;BW_{RX}=\gamma \cdot |\bar{G}|\cdot FOV$$

with *BW*_*RX*_ being the imaging bandwidth. This condition limits the maximum flip angle (*α*_*max*_) proportional to the maximum RF excitation field (*B*_1;*max*_) and inverse proportional to the imaging bandwidth according to *α*_*max*_ = 2 · *γ* · *B*_1,*max*_/*BW*_*RX*_. For example, assuming *B*_1,*max*_ = 15 μT and *BW*_*RX*_ = ±62.5 kHz, the maximum flip angle is limited to *α*_*max*_ ≈ 3.7°.

For a sufficiently large number of uniformly distributed spokes the effective excitation profile can, to a good approximation, be assumed to be spherically symmetric. However, inconsistent excitation dependent on the readout direction leads to blurring which increases with distance from isocentre. This can be corrected to a limited extent by correcting each spoke by the effective excitation profile as described by Grodzki et al. [48]. Alternatively, shaped RF pulses can be used to improve excitation uniformity in the presence of the readout gradient, but at the expense of lower maximum flip angle for the same B_1_ amplitude, as well as high demands on the time resolution of the RF transmit system [50]. It is also possible to reduce the excitation profile effects by performing RF excitation with reduced gradient amplitude and then increasing the amplitude for data acquisition, resulting in hybrid ramp sampling [51–53]. While this method enables excitation with higher flip angles, it will increase the acoustic noise due to increased gradient switching [51]. The acquisition will also be more sensitive to eddy currents from ramp-sampling, thus requiring gradient calibration for accurate image encoding [53].

**Fig. 2:**
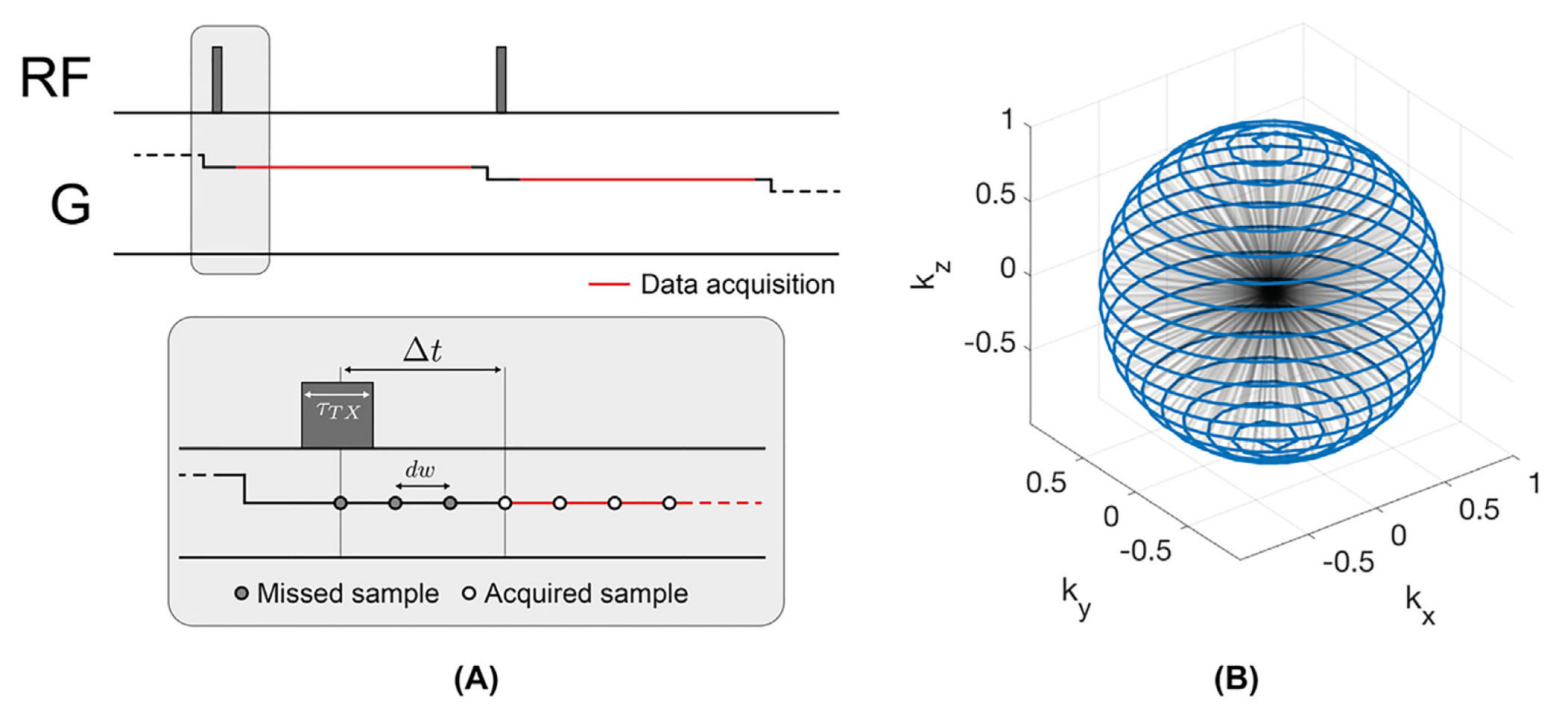
(**A**) Simplified ZTE pulse sequence diagram with two spokes, showing RF excitation and the gradient waveform on one axis together with magnification of the RF excitation part of the spoke, showing the dead-time gap Δ*t* after RF excitation. (**B**) 3D view of spoke distribution in k-space with the endpoints of each spoke connected by the blue line.

When RF excitation is applied in the presence of the readout gradient, k-space encoding starts immediately (hence TE = 0). Given the finite and non-zero switching time between RF transmit and receive mode, there is always a small dead-time gap (Δ*t*) as shown in Fig. 2A in the beginning of each spoke, resulting in a number of missed data points. This translates to a spherical region in the centre of k-space without acquired k-space samples. The number of radial samples missed (*n*_Δ_) during the dead-time gap (Δ*t*) can be estimated as

(3) $$n_{\text{Δ}}=\frac{\text{Δ}t}{dw}.$$

Several factors affect the dead-time gap Δ*t*, including the RF excitation pulse width (*τ*_*TX*_), as well as system-specific delays for the readout filter, and it is therefore difficult to estimate Δ*t* without detailed knowledge of the MR system [38]. As an example, with Δ*t* = 20 μs and BW_*RX*_ = ±62.5 kHz, the dead-time gap results in *n*_Δ_ ≈ 2.5 missed centre k-space samples.

A limited number of missing samples can be recovered during reconstruction using linear algebra, provided that symmetric spokes have been acquired, and that the imaging object is of finite support (i.e., imaging FOV fully encompasses the MR active object) [49,54]. For larger dead-time gaps there are several methods that acquire additional data to replace the missing samples similar to keyhole imaging [55], including: WASPI (Water- And fat-Suppressed proton Projection MRI) [56], PETRA (Pointwise Encoding Time reduction with Radial Acquisition) [57] and HYFI (Hybrid Filling) [58]. WASPI acquires a second radial acquisition with reduced gradient strength; resulting in fewer missed samples, while PETRA acquires the missing samples pointwise on a Cartesian grid. HYFI combines both pointwise encoding and radial projections with different gradient strength. We note that the name WASPI actually derives from a specific application of the ZTE sequence described in the original paper by Wu et al. [56], and not the reduced gradient amplitude approach to dead-time gap filling with which it has now become synonymous.

### 2.2 Silent 3D radial k-space sampling

Sampling of the free induction decay (FID) signal in the presence of a constant readout gradient naturally leads to a 3D centre-out radial k-space sampling scheme. By updating the readout direction in between excitations, a full spherical k-space is sequentially encoded, as shown in Fig. 2B. For a cubic image matrix size of size *N* × *N* × *N*, each spoke contains *N*_*pts*_ = *N*/2 sampling points. The number of spokes required to fulfil the Nyquist criterion (*N*_*Nyquist*_) at a maximum k-space radius is determined by the surface area of the k-space sphere, i.e., *N*_*Nyquist*_ = *πN*^2^ [59]. For equidistant radial sampling, the density decreases inversely proportional to the squared radius. Accelerating a 3D radial acquisition through angular undersampling, i.e., reduction of the number of spokes, will reduce the SNR and produce undersampling artefacts manifesting as streaking.

The non-selective excitation in ZTE will excite spins outside the FOV, potentially including plastic materials such as the RF coil and patient table [37,60]. To avoid aliasing of such signals, radial over-sampling is used to push the aliasing-sphere outside the imaging FOV [38]. Radial oversampling, resulting in a larger encoded FOV, is also essential for algebraic reconstruction of the deadtime gap as it ensures that the object has finite support in image space [49].

The essential features which render the ZTE pulse sequence silent are 1) constant gradient FID readout and 2) minimal updates of the readout direction (i.e., not readout amplitude) in between repetitions. This does not mean that all ZTE acquisitions are necessarily silent, but rather that a ZTE pulse sequence can operate within these constraints. For instance, the ZTE BLAST sequence shown in Fig. 1B, is not silent due to large gradient steps between readouts. The small change in gradient direction between spokes in RUFIS, on the other hand, enables silent acquisition. For complete 3D k-space coverage this can be achieved by arranging the spokes in a spiral pattern, as shown in Fig. 2B [61]. The acoustic sound pressure and frequency spectrum produced by a ZTE sequence depend on scan parameters such as the readout bandwidth, TR and number of spokes in the trajectory, since these will affect absolute gradient amplitude, the duration of each spoke, and the gradient steps between spokes. With commonly used scan parameters for ZTE, the acoustic noise typically stays within 5 dB of ambient noise levels, as shown in Table 1 which summarizes acoustic noise measurements from published studies using ZTE for silent imaging.

There are several imaging pulse sequences in the literature that can be used for silent ZTE imaging, including RUFIS, PETRA, WASPI, and Looping Star. For simplicity, and to clarify that our descriptions generalise across these methods, for the remainder of this paper we use “ZTE” as an over-arching term for any FID pulse sequence with a TE = 0 FID readout, which also features small gradient steps between readouts to allow near-silent data acquisition.

### 2.3 Native ZTE image contrast

The contrast behaviour of an RF spoiled steady-state ZTE sequence is similar to that of a spoiled gradient echo (SPGR) acquisition (often also referred to as GRE, FLASH, or Fast Field-Echo) [75]. In an SPGR acquisition, the longitudinal steady-state magnetization can be described by the following equation

(4) $$\begin{array}{l} M_{z,SPGR}=PD\cdot E_{2}^{*}\cdot \frac{\left(1-E_{1}\right)}{1-E_{1}\cdot \cos(\alpha )}\\ \;\text{with}\;E_{1}=e^{-\frac{TR}{T_{1}}},E_{2}^{*}=e^{-\frac{TE}{T_{2}}} \end{array}$$

with the contrast depending on T_1_, T_2_*, the proton density (PD) and the flip angle *α*. When *TE* = 0, T_2_* decay is reduced to a minimum, i.e., $E_{2}^{*}=1$, resulting in heightened sensitivity to tissues with very short T_2_, such as cortical bone [76], or myelin [77]. The TR is very short in ZTE, as it is determined only by the readout duration, and the flip angles that can be achieved are typically limited to just above the Ernst angle, unless a very low imaging bandwidth is used [67]. Hence Eq. (4) can be simplified through a first order approximation (i.e., TR ⪡ T_1_, and α ⪡ 1 rad) as

(5) $$M_{z,ZTE}\approx \frac{PD}{1+\frac{T_{1}}{TR}\cdot \frac{\alpha^{2}}{2}}$$

Native ZTE image contrast is therefore typically PD weighted with some T_1_ saturation. To increase contrast, practical implementations of ZTE typically split up the acquisition into segments to allow for contrast preparation (see Section 3.1), with the gradients being ramped slowly before and after each segment to avoid acoustic noise.

### 2.4 Image artefacts

Imaging with ZTE has several advantages besides being silent. The low gradient switching rate in ZTE, which makes the acquisition silent, also reduces eddy currents. With a TE = 0, there is no time for phase accumulation before the readout, resulting in reduced flow and motion artefacts [45]. However, ZTE is still vulnerable to phase accumulation during the readout, originating from off-resonance effects (e.g., main magnetic field inhomogeneity, tissue susceptibility and fat–water chemical shift). For clinical evaluation of ZTE images, the differences in appearance of off-resonance artefacts, compared to Cartesian acquisitions, are important to consider, as the artefacts otherwise could be misinterpreted as pathology [78]. Chemical shift off-resonance effects can be addressed by using a pixel bandwidth larger than the fat–water chemical shift (i.e., 430 Hz at 3T), and additionally mitigated using a k-space based in-phase and out-of-phase ZTE image decomposition as described by Engström et al [79]. Alternatively, fat saturation pulses can also be used [64,80].

**Fig. 3:**
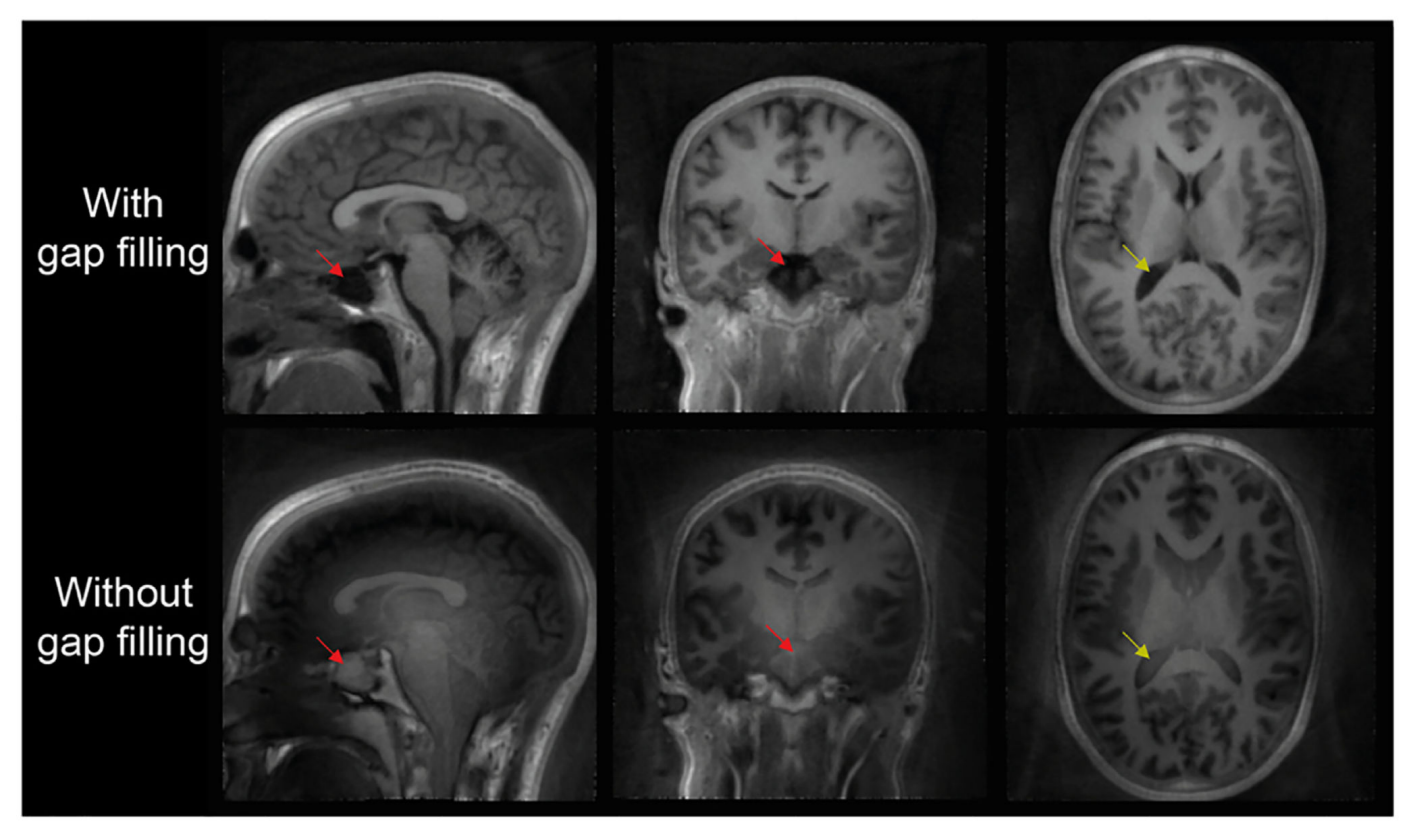
Example of a dataset reconstructed with and without WASPI to fill the centre of k-space. A clear low spatial frequency artefact appears across the image without WASPI, resulting in erroneous image contrast, especially visible in the lateral ventricles (yellow arrows) and the sinuses (red arrows). Data were acquired with a readout bandwidth of ±31.25 kHz.

A unique feature of ZTE imaging is the dead-time gap, as discussed in Section 2.1, which results in a spherical region in the centre of k-space without acquired valid samples. Since the centre of k-space encodes low spatial frequencies, the resulting artefact manifests in the form of a slowly varying background signal, rolling off towards the edges of the image (see Fig. 3). The effect is strongest in the centre of the image, and most apparent in areas with image intensity close to zero, such as the lateral ventricles (yellow arrows), the sinuses (red arrows) and the background.

**Fig. 4:**
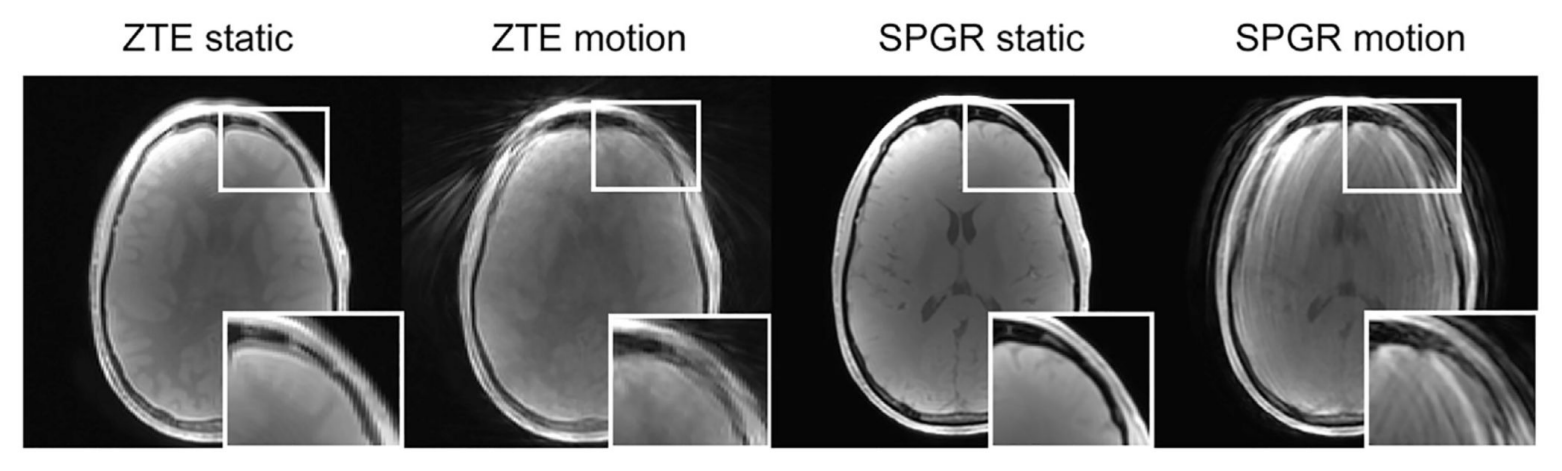
An example of how motion artefacts manifest as blurring and streaking in ZTE, while in Cartesian SPGR they produce ghosting in the phase-encode direction. Reproduced with permission from Ref. [82].

Radial k-space sampling is in general less sensitive to motion during data acquisition due to the repeated sampling, and hence averaging, of the k-space centre. Motion artefacts therefore appear as localized blurring and streaking instead of coherent ghosting across the imaging FOV seen in Cartesian acquisitions [81]. An example of head motion during data acquisition, comparing ZTE and a Cartesian sampled SPGR, is shown in Fig. 4.

## 3 ZTE pulse sequences and control of image contrast

The previous section described the basic ZTE pulse sequence and its native contrast behaviour. Because of its low flip angle RF excitation and effective TE = 0, the native PD and T_1_-weighted SPGR signal response contains minimal contamination from T_2_* relaxation, susceptibility artefacts, diffusion, and flow effects (see Eq. (4) and (5)). However, to enable use of ZTE in clinical settings, additional contrasts beyond PD and T_1_ are required. The following section describes modifications of the ZTE pulse sequence to encode additional contrasts via magnetization preparation and gradient echo refocusing.

### 3.1 Magnetization prepared ZTE

Magnetization prepared FLASH, as originally described by Haase [83], forms a powerful method to extend the contrast range of SPGR-type sequences beyond native PD and T_1_ weighting. For this, the acquisition is divided into segments, each segment starting with a magnetization preparation (MP) module to modify the longitudinal magnetization to contain a desired contrast weighting (e.g., T_1_, T_2_, MT, or diffusion), followed by a certain number of low-FA, short-TR SPGR acquisitions. MP-ZTE offers additional advantages in terms of being silent, TE = 0, and fast scanning with short TR where most of the time is used for image encoding. In a segmented ZTE acquisition, the longitudinal magnetization of spoke *i* within a segment can be expressed as [84]

(6) $$\begin{array}{l} M_{z,i}=\underset{Decay}{\underset{︸}{M_{prep}\cdot \beta^{i}}}+\underset{Recovery}{\underset{︸}{M_{z,SPGR}\cdot \left[1-\beta^{i}\right]}},\;\text{with}\;\beta \\ =E_{1}\cos\alpha ,\;E_{1}=e^{-\frac{TR}{T_{1}}}\;\forall i=\{0\ldots N-1\} \end{array}$$

Here *M*_*prep*_ is the longitudinal magnetization at the beginning of the segment, which is weighted by the MP and potential T_1_ weighting from incomplete recovery between segments. Eq. (6) shows that the information from the MP is encoded only in the decay term, where it is modulated by T_1_ relaxation. For large numbers of spokes (and/or high FA and long TR), *M*_*z*,*i*_ converges towards the steady-state SPGR signal given by Eq. (4) and the decay term (containing the MP weighting) vanishes.

In a Cartesian MP-SPGR acquisition, the first k-space line of each segment can be acquired close to the centre of k-space, i.e., centric ordering, such that the MP weighting dominates the image contrast [85]. In a ZTE acquisition where all readouts, i.e., spokes, originate at the centre of k-space, the image contrast is given by the average signal acquired by all *N* spokes in a segment, obtained by evaluating the geometric sum of Eq. (6) which yields [67]

(7) Mzseg=Mprep⋅f(N,β)︸Decay+Mz,SPGR⋅[1−f[N,β]]︸Recoveryf(N,β)=1N⋅1−βN(1−β), with β=E1cosα

For a single spoke (*N* = 1), *f* = 1 and the acquired signal is equal to the prepared magnetization. For a large number of spokes (i.e., *N*⟶∞), *f* ⟶ 1 and the acquired signal converges towards the steady state magnetization *M*_*z*,*SPGR*_.

There are several methods to reduce the influence of undesired T_1_ weighting in MP-ZTE. Reducing the number of spokes per read-out segment will minimize T_1_ weighting from the recovery term in Eq. (6), but at the expense of increased acquisition time as it would increase the number of preparation periods required. Hsu and Lowe proposed a method called *eliminative averaging*, achieved by combining two volumes where the prepared magnetization is *M*_*prep*,1_ = *M*_*prep*_ and *M*_*prep*,2_ = −*M*_*prep*_[84], effectively removing the recovery term and preserving the prepared magnetization. T_1_ weighting in the decay term in Eq. (6) can be corrected using a single T_1_ assigned to the whole object for a global correction of the k-space data [84]. An alternative method is to apply a k-space filter which increases the relative weighting in the centre of k-space to spokes acquired early in the segment, thus increasing the contribution to the main contrast from these spokes [86], similar to a Cartesian centre phase-encode ordering scheme.

In the applications outlined in Section 4, different types of MP will be described, which produce different contrasts, but all using the same concept of MP followed by one or more ZTE segments. Unless the preparation module includes strong gradients, as for diffusion preparation, the acoustic noise is not increased by MP, and is in fact typically reduced even further, as many MP methods require a delay period for T_1_ recovery.

### 3.2 Multi-echo gradient refocused ZTE

Analogous to multi-echo GRE or UTE, it is possible to design a multi-echo ZTE sequence by refocusing the excited FIDs to produce a gradient echo with T_2_* contrast. Conventionally, bipolar gradients are used for signal refocusing in gradient echo acquisitions, which results in loud acoustic noise from rapid gradient switching. Quiet gradient refocusing with minimal gradient switching can be achieved, as demonstrated with the Looping Star pulse sequence [71] where multiple FID signals are generated and gradient refocused in a looping, time-multiplexed, manner. Similarly, multi-echo ZTE can also be achieved using the BURST technique, albeit at the expense of higher acoustic noise [70]. In this section we will first describe the Looping Star pulse sequence followed by a brief description of ZTE-BURST.

#### 3.2.1 Looping Star

**Fig. 5:**
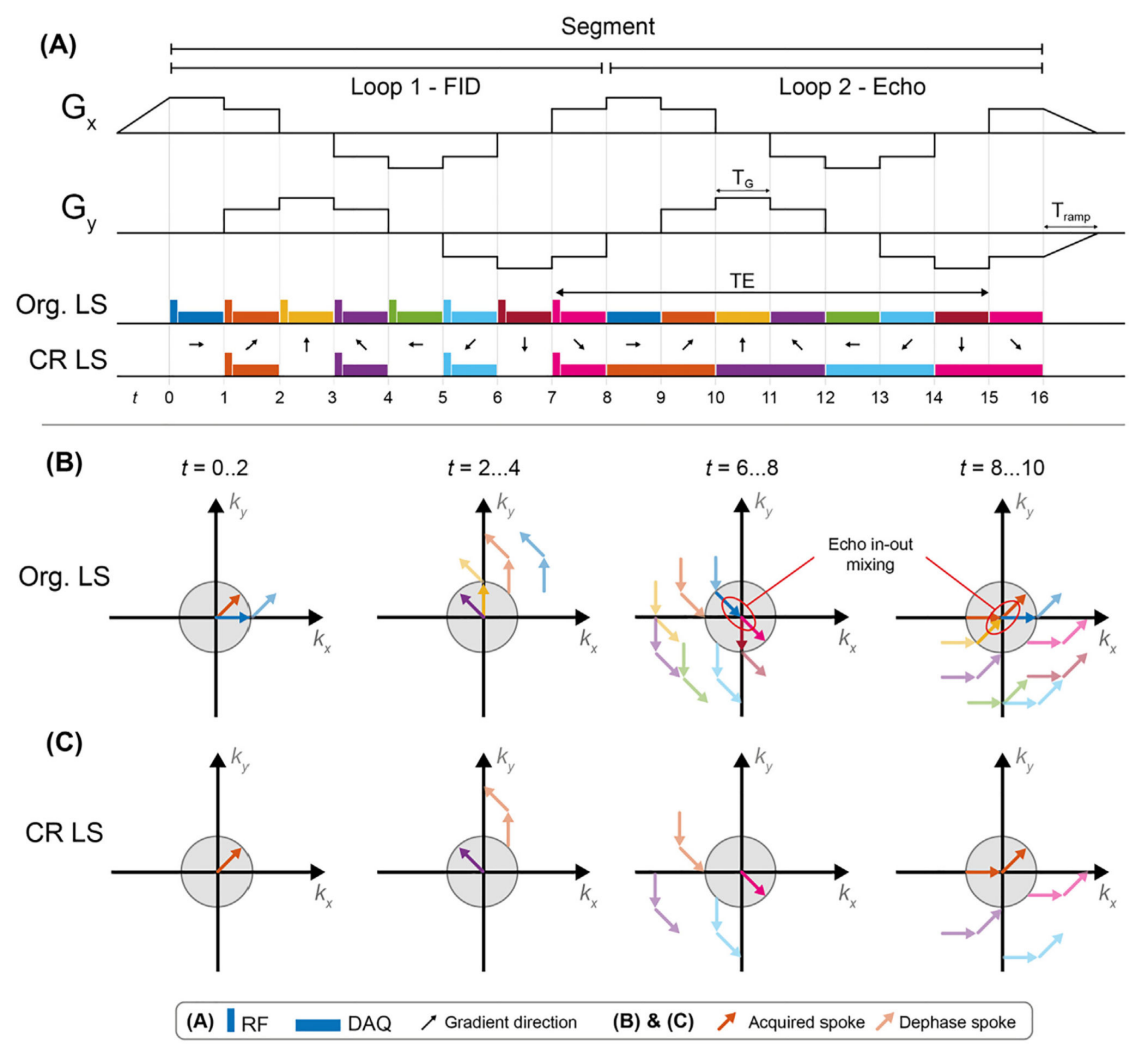
(**A**) Gradient waveform structure of a Looping Star sequence with 8 spokes per loop (*N*_*SPL*_) and 2 loops (*N*_*Loops*_), showing the RF and data acquisition (DAQ) scheme for the original (Org.) and coherence resolved (CR) versions of Looping Star. (**B**) and (**C**) illustrates the spin coherences for the two versions of Looping Star at four different timepoints during the sequence. The grey shaded region indicates nominal field of view in k-space as defined by the desired image resolution; coherences outside this region (faded arrows) are considered to be dephased and not contributing to the image.

The Looping Star pulse sequence uses a time-multiplexed gradient refocusing scheme to produce T_2_*-weighted gradient echoes, as shown in Fig. 5 [71]. The gradient amplitude is updated directly between spokes, to ensure quiet operation, similar to RUFIS. The k-space trajectory is designed such that each coherence follows a looping trajectory in k-space and periodically refocuses to the centre of k-space to form equidistant gradient echoes.

The acquisition is divided into segments, where each segment encodes a plane in k-space. Multiple segments, rotated relative to each other, are acquired for full 3D k-space coverage. In the first loop of a segment (Fig. 5A), a number of coherences are produced by gradients in different directions, each of which encodes a radial centre-out FID spoke, similar to standard ZTE. In the second loop, the same gradient waveform is then repeated, but without application of RF pulses, to refocus the magnetization and produce T_2_*-weighted gradient echoes (Fig. 5B). Since the magnetization is refocused in a continuous loop, the change in gradient direction and thus acoustic noise can be kept small. The number of spokes acquired per loop (*N*_*SPL*_), together with the duration of each spoke (*T*_*G*_), governs the acoustic noise and also determines the echo time (*TE*_*LS*_) according to

(8) $$TE_{LS}=N_{SPL}\cdot T_{G}$$

The overall acquisition time for a volume (*T*_*acq*_), equivalent to

(9) $$T_{acq\;}=\left(T_{G}\cdot N_{SPL\;}\cdot N_{Loops\;}+2\cdot T_{ramp\;}\right)\cdot N_{Seg\;}$$

the TR in an fMRI experiment, is given by

where *N*_*Loops*_ is the number of loops (i.e., FID plus number of echoes), and *N*_*Seg*_ is the number of segments which is determined by the level of undersampling in the acquisition. Gradient ramp-up time (*T*_*ramp*_) is typically between 2 and 5 ms to ensure silent operation.

The original version of Looping Star shown in Fig. 5A as Org. LS, corresponding to that initially published by Wiesinger et al., suffers from mixing of the inwards refocusing and outwards dephasing coherences, known as echo-in/echo-out mixing [71] (see Fig. 5B). These signals can be separated through k-space filtering or RF phase cycling, but at the expense of reduced image quality, or increased scan time. In a further development of the pulse sequence, this temporal overlap problem was resolved via separation in time by performing RF excitation only every other spoke, hereafter referred to as coherence resolved Looping Star (CR LS), as shown in Fig. 5A and Fig. 5C. This reduces the number of excited coherences by half, and results in refocusing of spokes with twice the length of the FID, with a piecewise linear trajectory, crossing the centre of k-space in the middle of the readout (see orange arrows in Fig. 5C t = 8…10). The gradient echo spokes are thus sampling along a “curved diameter” without overlap between coherences in the nominal k-space sphere (compare Fig. 5B and C at t = 8…10).

#### 3.2.2 ZTE-BURST

**Fig. 6:**
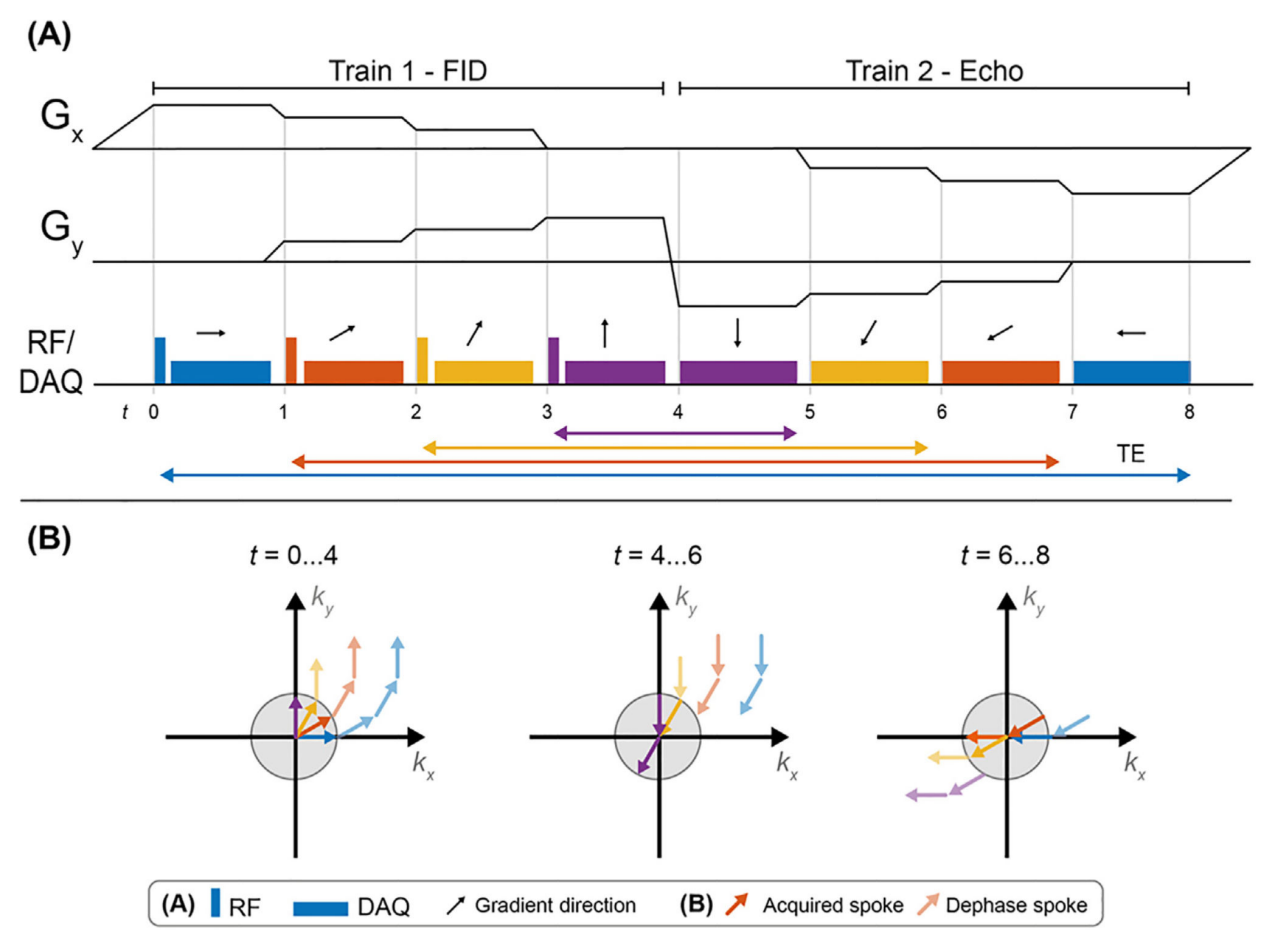
(**A**) Pulse sequence diagram of a single echo gradient refocused ZTE-BURST sequence, with the TE for each coherence indicated by the correspondingly coloured arrow. (**B**) Visualization of the evolution of the four coherences through the first and second train. The grey circle illustrates the nominal coverage in k-space as determined by the image resolution. K-space sampling is here illustrated in 2D for simplicity, while in practice it is performed in 3D.

The BURST pulse sequence consists of a series of short, low flip angle, RF pulses applied in the presence of a gradient, which can subsequently be refocused using gradients or refocusing RF pulses (analogous to gradient-echo and spin-echo refocusing techniques) to produce a series of gradient or spin echo signals [87]. Schulte et al. combined the concept of BURST imaging with ZTE [70], as shown in Fig. 6. The sequence consists of trains of *N*_*SPT*_ spokes where in the first train, RF pulses are applied to produce *N*_*SPT*_ FIDs, which are encoded separately. In the second train, the RF is turned off and the trajectory is rewound, producing *N*_*SPT*_ gradient echoes as the FIDs are refocused, resulting in T_2_* weighting that varies between echoes.

The acoustic noise produced by ZTE-BURST is slightly higher than Looping Star due to rapid gradient switching between the trains. Schulte et al. measured 75.8–78.2 dBA for the ZTE-BURST sequence (depending on the settings), compared to a background scan room level of 66.6 dBA [70] (see Table 1).

## 4 ZTE for structural neuroimaging

### 4.1 T_1_ contrast mechanisms

Native ZTE imaging provides SPGR-type PD and T_1_ contrast weighting, as shown in Eq. (5). Using variable flip angle (VFA) imaging, this can be extended to quantitative PD and T_1_ mapping [88]. For a given TR, the degree of T_1_ weighting is limited by the maximum achievable flip angle, which is determined by *B*_1,*max*_ and by the maximum RF pulse width (τ_*TX*_). To avoid slice profile effects, *τ*_*TX*_ is limited by the imaging bandwidth (*BW*_*RX*_), as described in Eq. (2). Within these constraints, Ljungberg et al. demonstrated VFA T_1_ mapping with RUFIS at 3T using low readout bandwidth (±7.8 kHz) to achieve high enough flip angles, in the case of that study 12° [67]. Their VFA ZTE acquisition produced T_1_ values very similar to standard SPGR acquisition, with equivalent reproducibility and repeatability.

Preliminary results have also demonstrated the utility of VFA T_1_ mapping with RUFIS across field strengths [89,90]. As T_1_ gets longer with increasing field strength [91], lower flip angles are required to obtain the same T_1_ contrast. However, higher field strengths typically require higher readout bandwidth to minimise chemical shift artefacts, and the maximum flip angle is thus reduced. In addition, increased B_1_ inhomogeneity at high field will reduce the effective flip angle further. On the other hand, at lower field strengths, VFA ZTE T_1_ mapping is better conditioned, as higher flip angles are possible with lower readout bandwidth requirements and better B_1_ uniformity.

In the context of VFA T_1_ mapping, Ljungberg et al. also demonstrated a B_1_ mapping technique using magnetization prepared ZTE [67,92], similar to the preconditioned TurboFLASH method by Chung et al. [93]. Ljungberg et al. used a train of ultrashort hard RF pulses, similar to those used in the ZTE readout, with a total flip angle of *α*_*prep*_ followed by a short readout segment and a T_1_ recovery period. The magnetization preparation will thus effectively encode $B_{1}^{+}$ as a global scaling in the image intensity, and a $B_{1}^{+}$ map can be calculated from a set of images with different *α_prep_*.

**Fig. 7:**
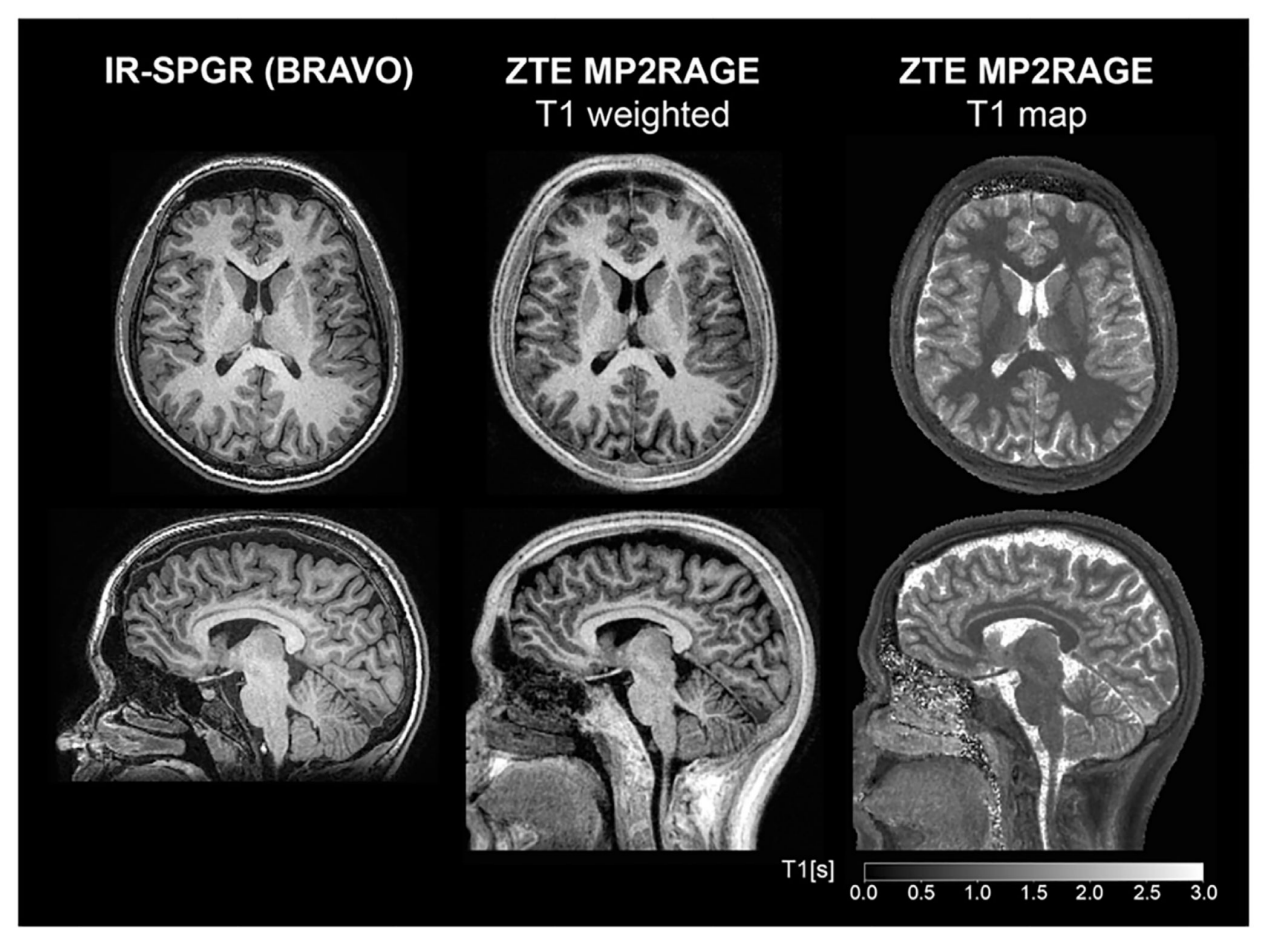
Comparison of anatomical T_1_-weighted imaging between Cartesian IR-SPGR using the GE BRAVO (Brain Volume imaging) sequence and ZTE acquired using the MP2RAGE formalism. T_1_ map is obtained from the ZTE-MP2RAGE acquisition. Acquisition parameters in Ref. [99].

**Fig. 8:**
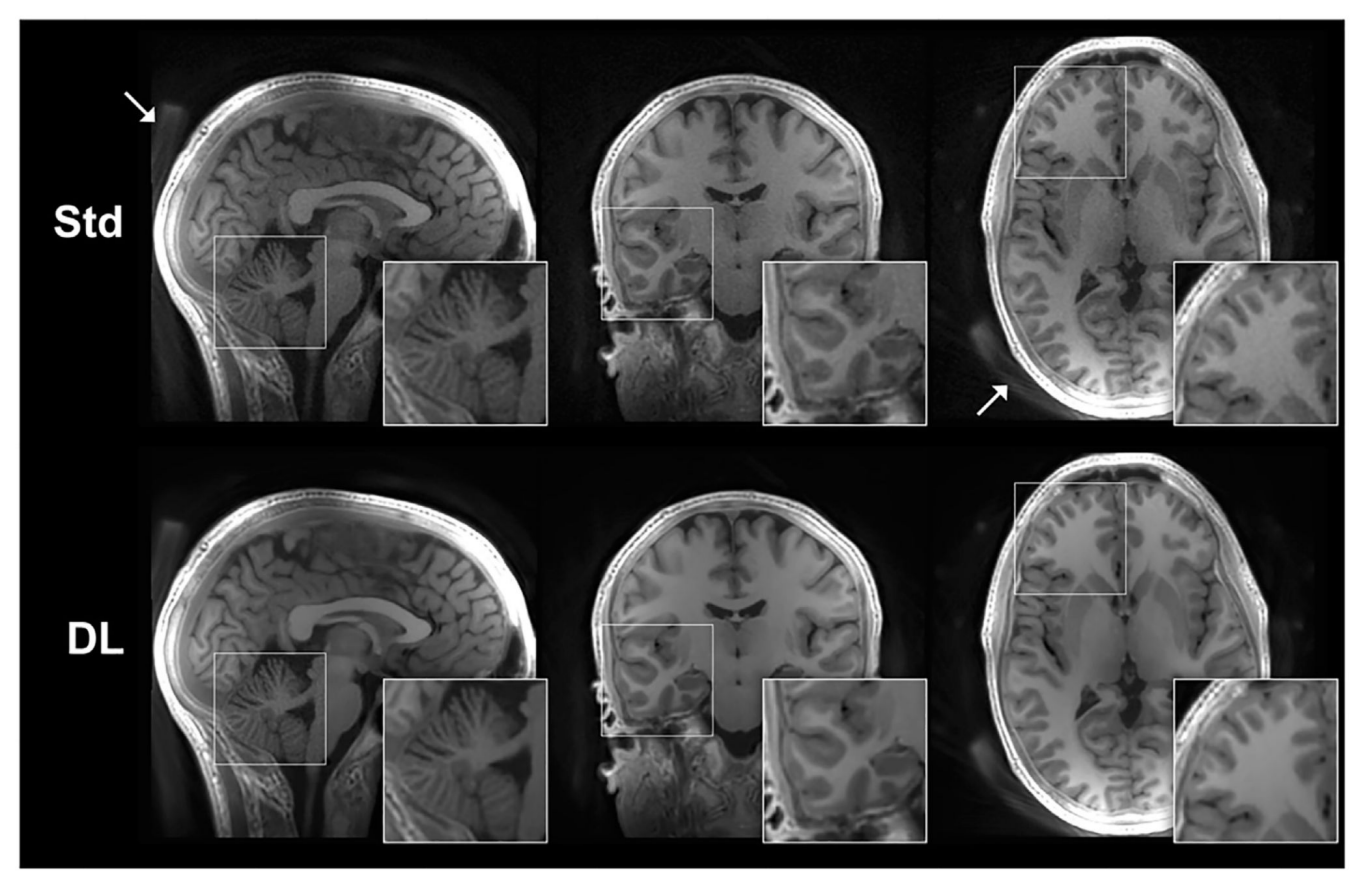
Example of IR-ZTE dataset reconstructed with and without Deep Learning denoising (DL vs. Std). With DL denoising, the image noise is clearly reduced while still maintaining image resolution and sharpness. White arrows indicate signal from the head rest.

An alternative method to obtain T_1_ contrast is to use an inversion or saturation pulse followed by a segmented ZTE readout, in analogy to the MPRAGE (Magnetization Prepared Rapid Gradient Echo) method [94]. Several studies have used inversion recovery (IR) prepared ZTE for T_1_-weighted imaging, with comparison to Cartesian IR prepared SPGR at both 3T [62,63,65,66,95] and 7T [64,80,96]. Applications of IR-ZTE at 7T have used interleaved fat saturation for improved image quality [64,80,97,98]. Similarly, ZTE can be adopted for the MP2RAGE formalism [99] to obtain a bias-field corrected image and quantitative T_1_ map, either as two separate acquisitions [100], or as a combined acquisition [101]. Fig. 7 shows an example of a ZTE-MP2RAGE acquisition, demonstrating its ability to produce images with excellent contrast between white and grey matter [101]. Published IR-ZTE results appear promising and demonstrate potential to become equivalent to current MPRAGE T_1_-weighted neuroimaging in terms of image contrast and resolution, especially with development of new image reconstruction techniques such as compressed sensing [102] and Deep Learning [103,104]. Fig. 8 shows an example of an IR-ZTE dataset reconstructed with and without Deep Learning denoising [103], demonstrating how image details are preserved while noise is reduced.

### 4.2 T_2_ contrast mechanisms

T_2_ contrast can be obtained with a ZTE sequence in a manner similar to previous work on T_2_-prepared FLASH [105] and MPRAGE [106,107]. A T_2_-preparation module typically consists of a tipdown pulse, putting the magnetization in the transverse plane where it relaxes with T_2_. A number of refocusing pulses are then applied, after which a tip-up pulse is performed, putting the magnetization back along the longitudinal axis with the desired T_2_ weighting [105,108]. For improved robustness to B_0_ and B_1_ inhomogeneity, adiabatic T_2_ preparation such as the mBIR4 pulse can also be used [109,110].

To minimize contribution from T_1_ saturation and maintain T_2_ contrast, a T_1_-recovery period is required after each segment. The segment should preferably be as short as possible to avoid T_1_ recovery during the readout, or alternatively eliminative averaging can be used. There are only a few examples of T_2_-weighted ZTE in the literature; most notably T_2_-prepared ZTE for BOLD fMRI at 3T [74] and 7T [111]. In both studies, each volume was encoded with 1024 spokes, separated into two segments with T_2_ preparation before each segment and a 500 ms T_1_-recovery period.

A T_2_-prepared ZTE acquisition can be accelerated with multiple interleaved T_2_-preparation pulses, giving a cumulative effect for each T_1_-recovery period [112], similar to a fast spin echo acquisition in which images with multiple TEs are acquired. While the contrast in such images is dependent on T_2_, as desired, there is also significant influence of T_1_ which makes the image appearance diverge from pure T_2_ contrast, and also makes T_2_ mapping challenging unless T_1_ in each voxel is known. It is also possible to obtain T_2_ weighting without preparation using spin echo ZTE-BURST, in which a pair of refocusing RF pulses is inserted between readout trains [70]. Since ZTE-BURST uses very short readout segments, the influence of T_1_ is small and so the sequence can be directly used for T_2_ mapping [70].

### 4.3 Multiparametric ZTE

By combining T_1_- and T_2_-preparation modules, T_1_, T_2_ and PD can be quantified simultaneously. The approach proposed by Wiesinger et al. [113,114] resembles the method proposed by Kvernby et al. for combined T_1_, T_2_, and PD mapping for cardiac and brain applications [115,116]. In the multi-parametric ZTE method of Wiesinger et al. [113,114], an inversion pulse is applied and several ZTE segments are collected to sample T_1_ contrast. As the steady state is approached, T_2_ preparation is applied and another ZTE segment, with combined T_1_ and T_2_ contrast, is acquired. The signal evolution during this transient acquisition can be calculated using the framework outlined in Section 3.1, and the quantitative values can be obtained either through curve fitting or dictionary methods.

**Fig. 9:**
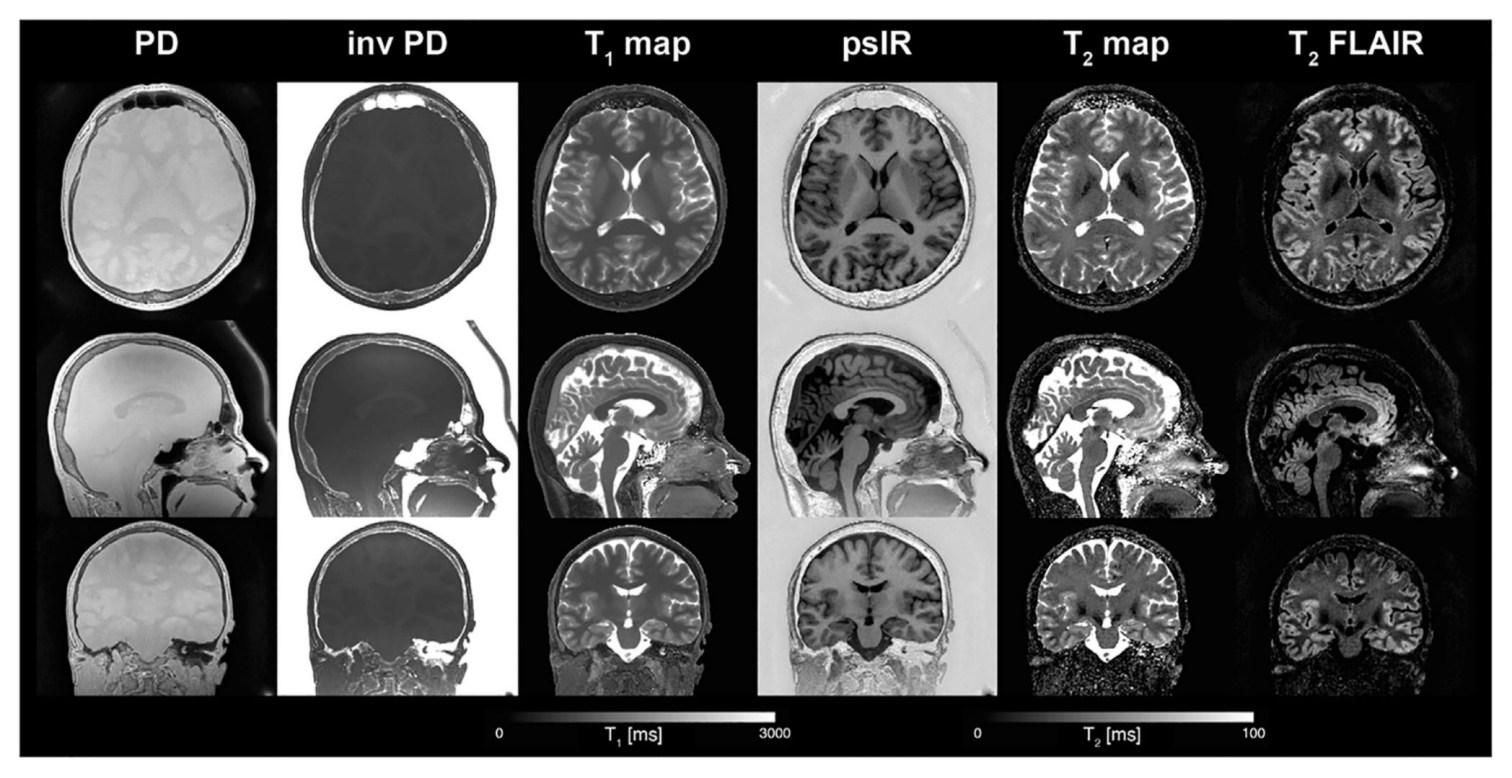
Quantitative PD, T_1_ and T_2_ maps obtained using a multi-parametric ZTE sequence together with synthetic contrast weighted psIR and T_2_ FLAIR images. Acquisition parameters: FOV = 20 × 20 × 16 cm^3^, resolution = 1 × 1 × 1 mm^3^, TR = 1.8 ms, BW_RX_ = ±31.25 kHz, TE _T2Prep_=80 ms, 256 spokes per segment. FA = 3° for multi-parametric part and FA = 1° for PD volume. Total acquisition time was 6:35 min. The T_1_ map and T_2_ map have been head masked using the PD volume. Abbreviations: **inv PD** – inverse PD, **psIR** – phase sensitive inversion recovery, **FLAIR** – fluid attenuated inversion recovery. Images have been cropped to head coverage. Fig. 10 shows the PD data reconstructed at twice the prescribed field of view. (Images generated with data from Ref [114]).

**Fig. 10:**
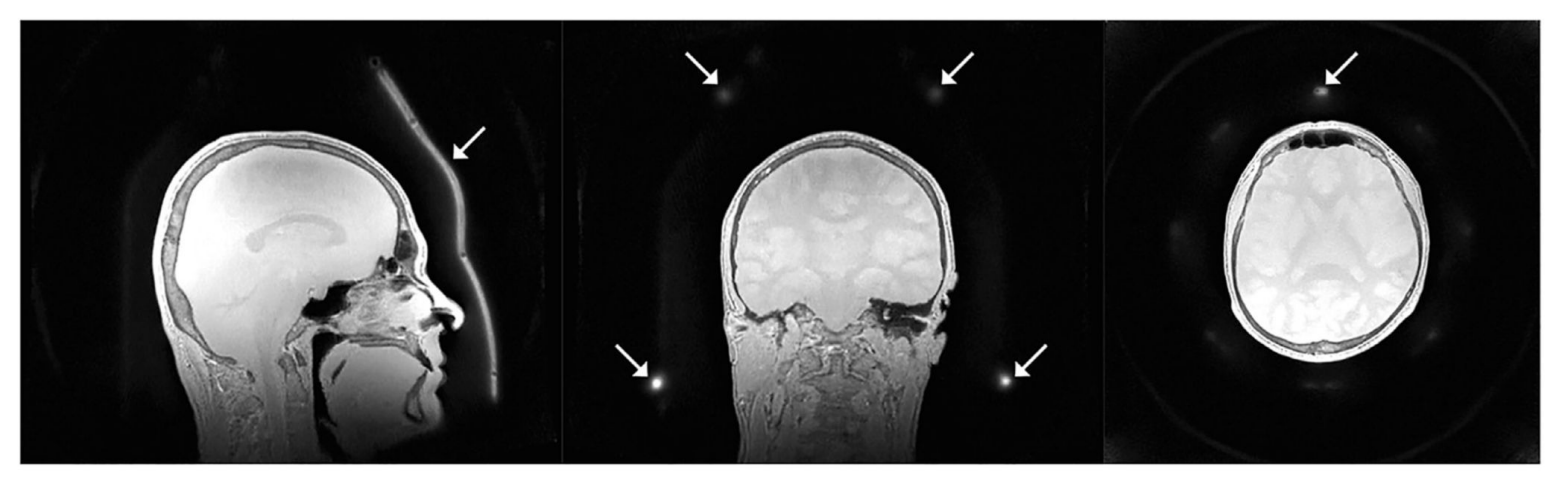
Proton density (PD) image from Fig. 9 reconstructed with twice the field of view, i.e., the fully encoded field of view from the twofold radial oversampling. White arrows highlight part of the receive coil only visible when reconstructed at twice the field of view.

Fig. 9 shows an example of quantitative T_1_, T_2_, and PD maps obtained with the multi-parametric ZTE sequence using three ZTE segments after the inversion pulse, followed by a T_2_-preparation module with TE = 80 ms [114]. A PD weighted volume was acquired separately and used in the fitting to improve the quantification. Synthetic phase sensitive IR (psIR) and T_2_-weighted fluid attenuated IR (FLAIR) images shown in Fig. 9 were calculated from the PD, T_1_ and T_2_ maps using an analytic signal equation, evaluated for each voxel. The data shown in Fig. 9 were collected using twofold radial oversampling, as commonly used in ZTE and discussed in Section 2.2, but cropped to show only the central portion of the resulting image. Radial oversampling enables reconstruction of a larger FOV than that prescribed, however. Fig. 10 shows the PD data from Fig. 9 reconstructed without this cropping, which results in an image with twice the prescribed FOV within which signal from the head coil, as indicated by the arrows, is clearly visible.

### 4.4 Diffusion contrast

**Fig. 11:**
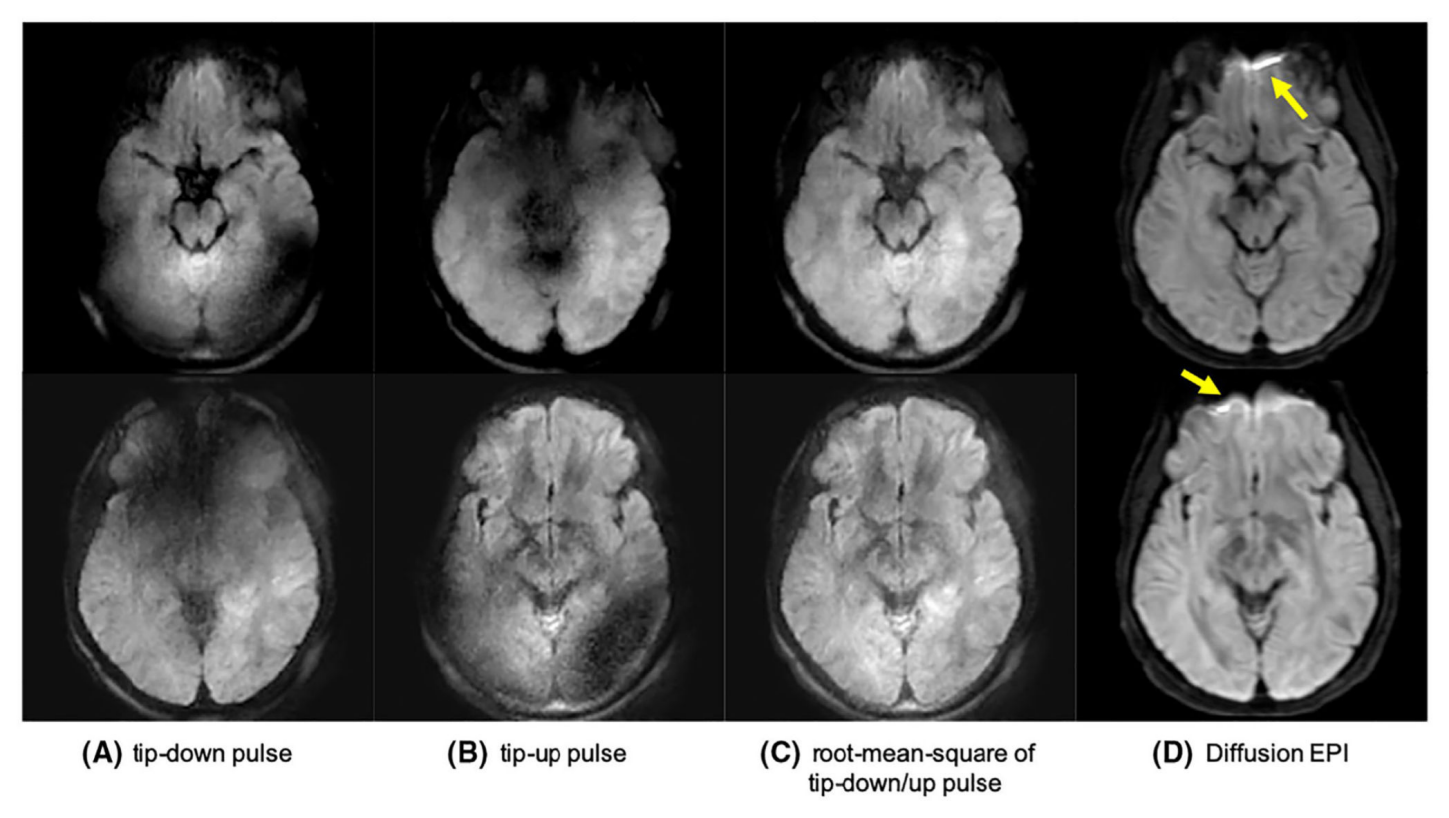
DW-ZTE (**A-C**) and DW-EPI (**D**) with b = 600 s/mm^2^. (A) and (B) demonstrate the eddy current artefacts, which are eliminated when combined in (C). Arrows highlights areas with distortion artefacts in DW-EPI that were not present in the DW-ZTE data. Reproduced with permission of John Wiley and Sons from Ref. [69], © 2019 International Society for Magnetic Resonance in Medicine.

Diffusion weighting (DW) can be achieved with a preparation module similar to that used for T_2_ preparation but with diffusion gradients added around the refocusing pulse. This approach has been previously demonstrated with Cartesian MPRAGE [117–119] and more recently using ZTE by Yuan et al. [69], who used sinusoidal diffusion gradients to reduce the acoustic noise. To remove the T_1_ saturation from the readout Yuan et al. used eliminative averaging, as proposed by Hsu et al. [84], while to correct for the T_1_ relaxation in the decay term in Eq. (6), they applied a global T_1_ correction, estimated from a WASPI acquisition. They also performed phase cycling of the tip up pulse to compensate for eddy currents, resulting in a minimum of 4 volumes acquired per diffusion encoding, b-value. The acquisition time per b-value was 3 min for an in vivo brain scan with 1.56 × 1.56 × 6 mm^3^ resolution, as shown in Fig. 11. Acoustic noise levels were measured to be only 3 dB above ambient (see Table 1).

Diffusion weighted ZTE has reduced off-resonance artefacts compared to EPI, shown with arrows in Fig. 11. Yuan et al. also reported examples of prostate and knee imaging where distortions using DW-ZTE were markedly reduced compared to standard DW-EPI. Similar improvements were seen in a study by Sandberg et al., where 39 paediatric patients underwent extremity MRI to compare DW-ZTE and DW-EPI in bone marrow, muscle and lesions [120]. DW-ZTE provided similar diffusion metrics to DW-EPI but in some cases with improved image quality due to reduced distortions.

### 4.5 Magnetization transfer

Magnetization transfer (MT) is an effective and time-efficient mechanism for generating strong contrast between white and grey matter by exploiting large amounts of broad-resonance protons in the lipids of myelin [121,122]. MT preparation can also be used to improve contrast in MRA experiments as it suppresses the signal in the tissue more than the signal from blood [123,124].

**Fig. 12:**
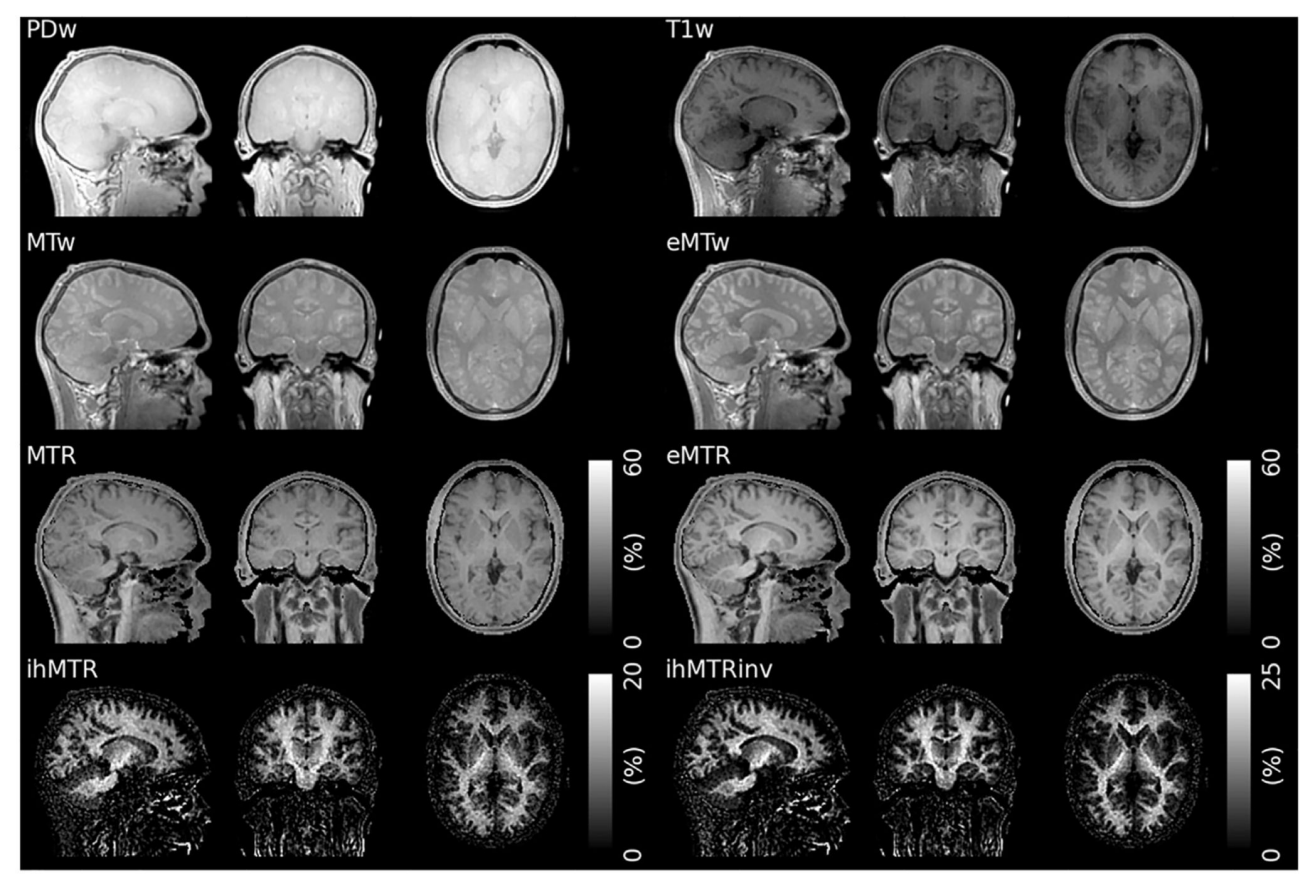
Examples of different MT contrasts acquired with a ZTE sequence. The ihMTR and ihMTRinv images show high sensitivity and specificity to myelin (Abbreviations: **PDw** – Proton Density weighted, T1w – T_1_ weighted, **MTw** – MT weighted, **eMTw** – enhanced MT weighted, with dual-sided saturation, **MTR** – Magnetization Transfer Ratio, **eMTR** – enhanced MT Ratio, **ihMTR** – inhomogeneous MTR, the difference between eMTR and MTR, **ihMTRinv** – inverse ihMTR using the T1w image as a reference instead of the PDw image). Reproduced with permission from Ref. [129] licensed under a CC BY 4.0 license. Figure has been cropped; original figure also contains a row with coefficient of variation.

Pulsed MT methods, in which an off-resonance preparation pulse is interleaved with a gradient-echo readout of a steady state acquisition [125], can easily be adapted to a segmented readout [126]. They are hence well suited for implementation with ZTE, as first demonstrated by Holmes et al. [127]. Wood et al. incorporated inhomogeneous MT (ihMT) [128] preparation into a ZTE sequence, showing high specificity to white matter in the brain, as illustrated in Fig. 12 [129]. The ihMT effect requires a material that can sustain dipolar order, and can as such be tuned for increased specificity to the properties of the semi-crystalline myelin sheath [130,131].

Grochowski et al. demonstrated use of MT prepared ZTE for anatomical imaging of the optic nerve at 7T using an adiabatic spectral inversion recovery pulse (ASPIR) for fat saturation, applied 1250 Hz off-resonance, which in addition to reducing the signal from fat also introduces MT contrast [132].

### 4.6 Angiography

Magnetic Resonance Angiography (MRA) contrast can be achieved by combining ZTE imaging with an Arterial Spin Labelling (ASL) preparation module [133]. Since ASL contrast is commonly produced by a series of low flip angle RF pulses, it does not increase the acoustic noise levels. Shang et al. demonstrated ZTE-MRA with less than 4 dB increase from ambient noise levels [68].

**Fig. 13:**
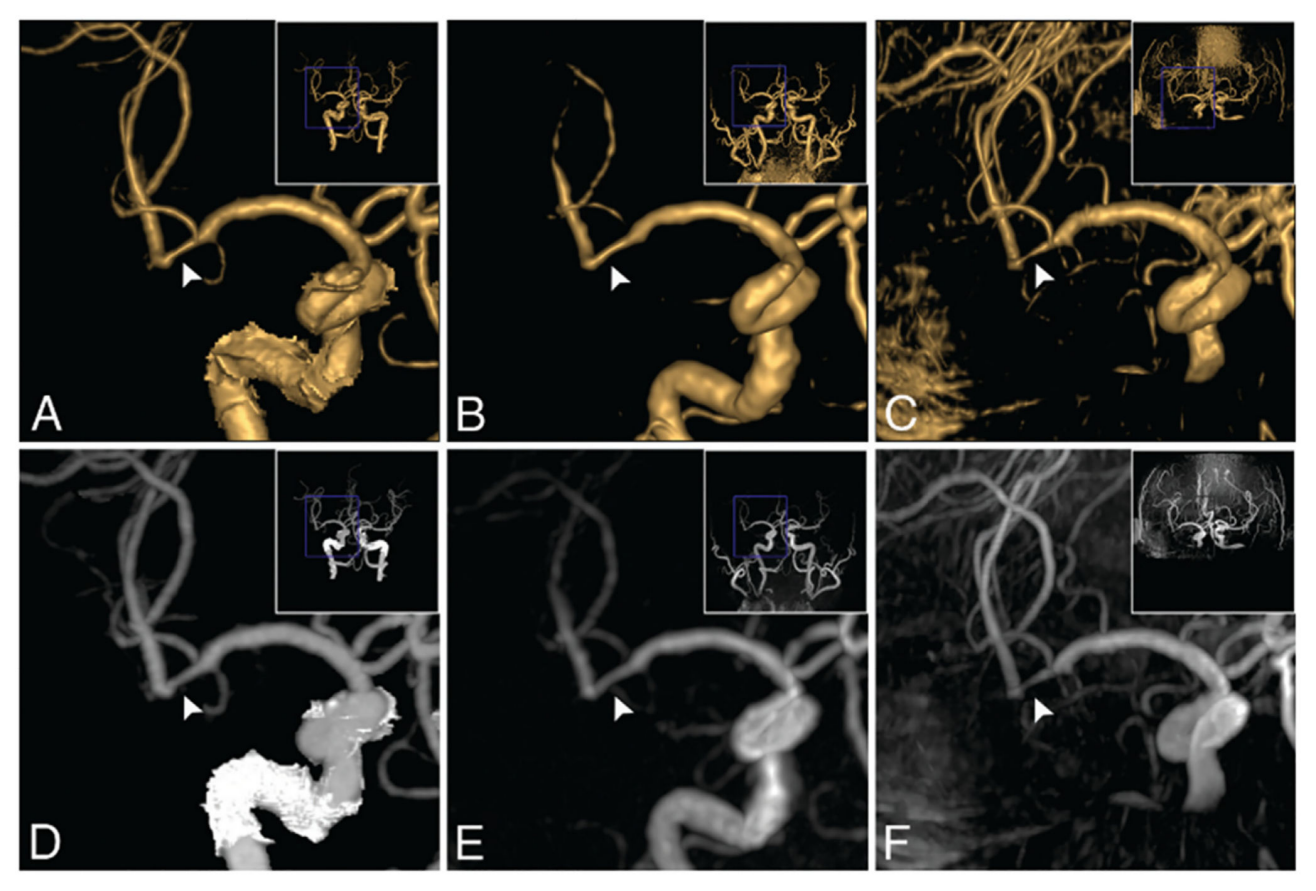
Example of ZTE-MRA (**B** and **E**) compared to computed tomography angiogram (CTA) (**A** and **D**) and TOF (**C** and **F**). Top row shows volume rendering and bottom row maximum intensity projection. Image shows a stenosis in a 74-year-old male patient. CTA estimated a 34% stenosis, ZTE estimated 32%, while TOF overestimated the stenosis to 72%. Republished with permission of American Society of Neuroradiology from Ref. [68]; permission conveyed through Copyright Clearance Center, Inc.

There are several potential benefits of using ZTE for MRA, besides reduced acoustic noise. Early work on RUFIS by Madio and Lowe demonstrated that RUFIS can image turbulent flow [45] and is capable of quantifying flow velocities in the presence of stenosis [134]. These results were corroborated in a phantom study, demonstrating better vascular display accuracy in the presence of stenosis with ASL-PETRA than with 2D and 3D Time Of Flight (TOF) angiography [135]. Further support to these results was provided in an in vivo study by Shang et al. where ZTE-MRA outperformed TOF imaging for assessment of stenosis and aneurysms in cerebrovascular diseases (see Fig. 13) [68]. Fujiwara et al. also demonstrated improved vessel contrast in the carotid artery with ZTE compared to 3D TOF [136].

Another advantage of ZTE-MRA is reduced artefacts around areas of magnetic susceptibility gradients, as demonstrated for DW-ZTE (Fig. 10), which is relevant for imaging around stents and coils in MRA. These artefacts are can be minimised by reducing the TE [137], and thus ZTE sequences have an advantage. Several studies have demonstrated improved vessel visualization around stents and coils with ZTE-MRA compared to conventional gradient echo based TOF-MRA [133,138–141].

The clinical utility of ZTE-MRA is still debated, however, with only a small number of studies published to date. Shang et al. performed a study on 68 patients with suspected cerebrovascular disorder, finding higher inter-modality agreement between ZTE-MRA and computed tomography angiography (CTA), than TOF and CTA [68]. However, in a study by Holdsworth et al. with 51 patients where ZTE-MRA was compared to 3D SPGR-TOF, the ZTE images were rated significantly lower across four categories (image blurring and SNR, lesion conspicuity, and diagnostic confidence), with only 48% of the ZTE-MRA, compared to 90% of SPGR-MRA, scans being judged to be of diagnostic quality [95].

### 4.7 Quantitative susceptibility mapping and T_2_*

Multi-echo gradient refocused ZTE can be used for quantitative susceptibility mapping (QSM) and T_2_* imaging using either Looping Star [71] or ZTE-BURST [70]. Both methods have demonstrated promising single-subject results, though the 0.8 mm resolution Looping Star QSM protocol was quieter (72.6 dBA) than the 1.00 mm ZTE-BURST protocol (75.8 dBA), both with an acquisition time of approximately 11.5 min.

**Fig. 14:**
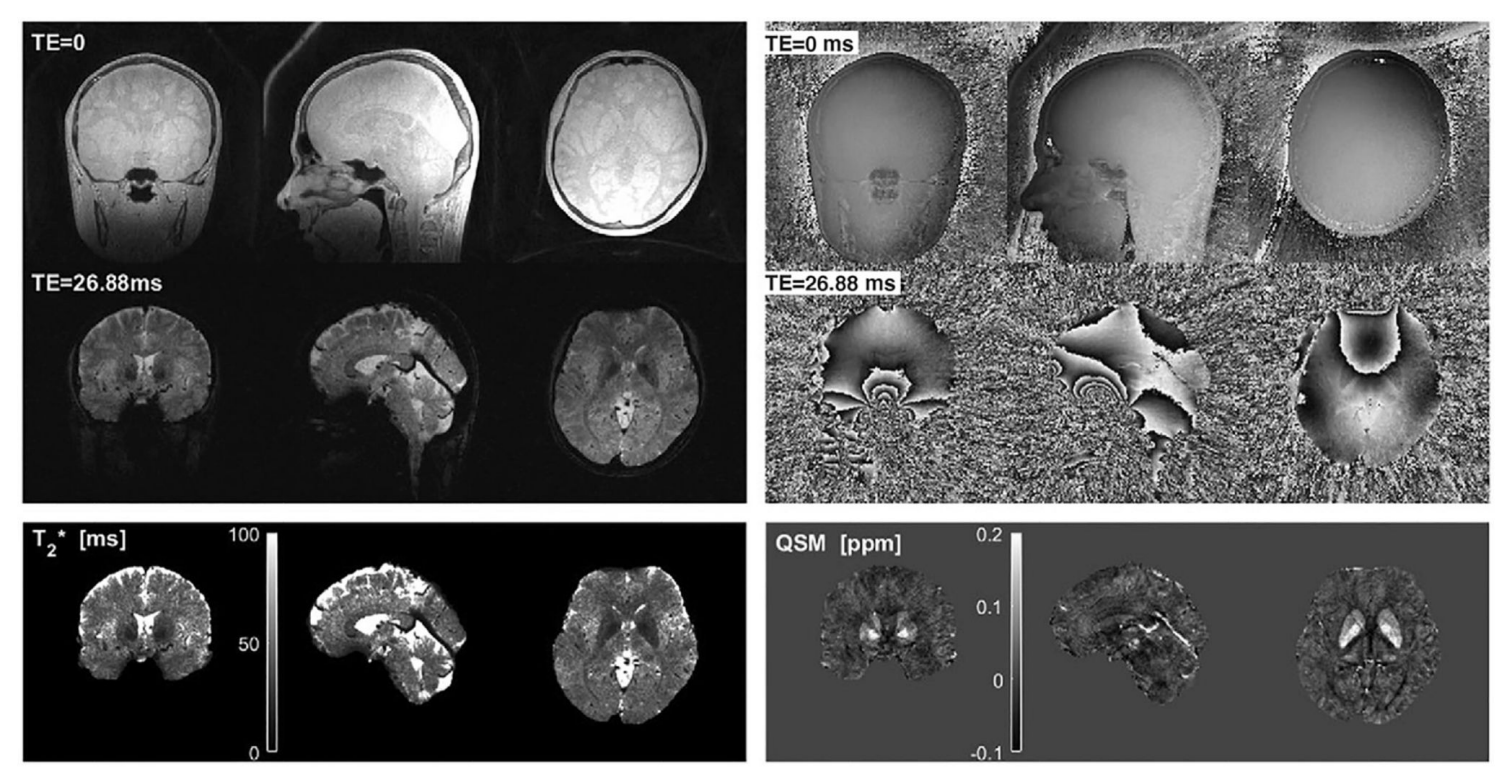
Example of T_2_* and QSM imaging with Looping Star. Reproduced with permission of John Wiley and Sons from Ref. [71] and adapted to highlight the TE in the right panel. © 2018 International Society for Magnetic Resonance in Medicine.

The TE in Looping Star and ZTE-BURST cannot be chosen arbitrarily, as shown in Eq. (8), and the minimum TE may need to be longer than that of a typical Cartesian acquisition in order to maintain silent operation. In the limiting case with two spokes per loop in Looping Star, the sequence essentially uses bi-polar gradients and is therefore no longer silent. The number of spokes per loop required to maintain silent operation depends on the gradient strength, as this will determine the absolute change in gradient amplitude. Wiesinger et al. demonstrated Looping Star QSM acquired with TE = 26.88 ms using 12 spokes per loop (Fig. 14) which resulted in acoustic noise of 72.6 dBA; for comparison, their 3 mm resolution fMRI acquisition using 32 spokes per loop measured 66.9 dBA [71].

As previously mentioned in the context of DW-ZTE, an advantage of multi-echo ZTE compared to conventional gradient refocused acquisitions is reduced geometric distortions from variations in magnetic susceptibility and reduced artefacts from motion and eddy currents. The high SNR FID (TE = 0) image can be used as a distortion-free reference image for spatial normalization of the gradient echoes, as well as an additional data point in quantitative T_2_* mapping and QSM. Furthermore, the gradient echo spokes can detect fluctuations in the B_0_ field as they sample the centre of k-space, as demonstrated by Wiesinger et al. [71]. Finally, the interleaved acquisition strategy used in Looping Star also enables reconstruction of motion navigators during the acquisition for retrospective motion correction.

### 4.8 Ultra-short T_2_: Bone and myelin imaging

ZTE pulse sequences can capture the MR signal from tissues with ultra-short T_2_, less than 1 ms [142], which typically are considered MR invisible [143]. In neuroimaging, ultra-short T_2_ tissues of interest mainly include bone and myelin.

**Fig. 15:**
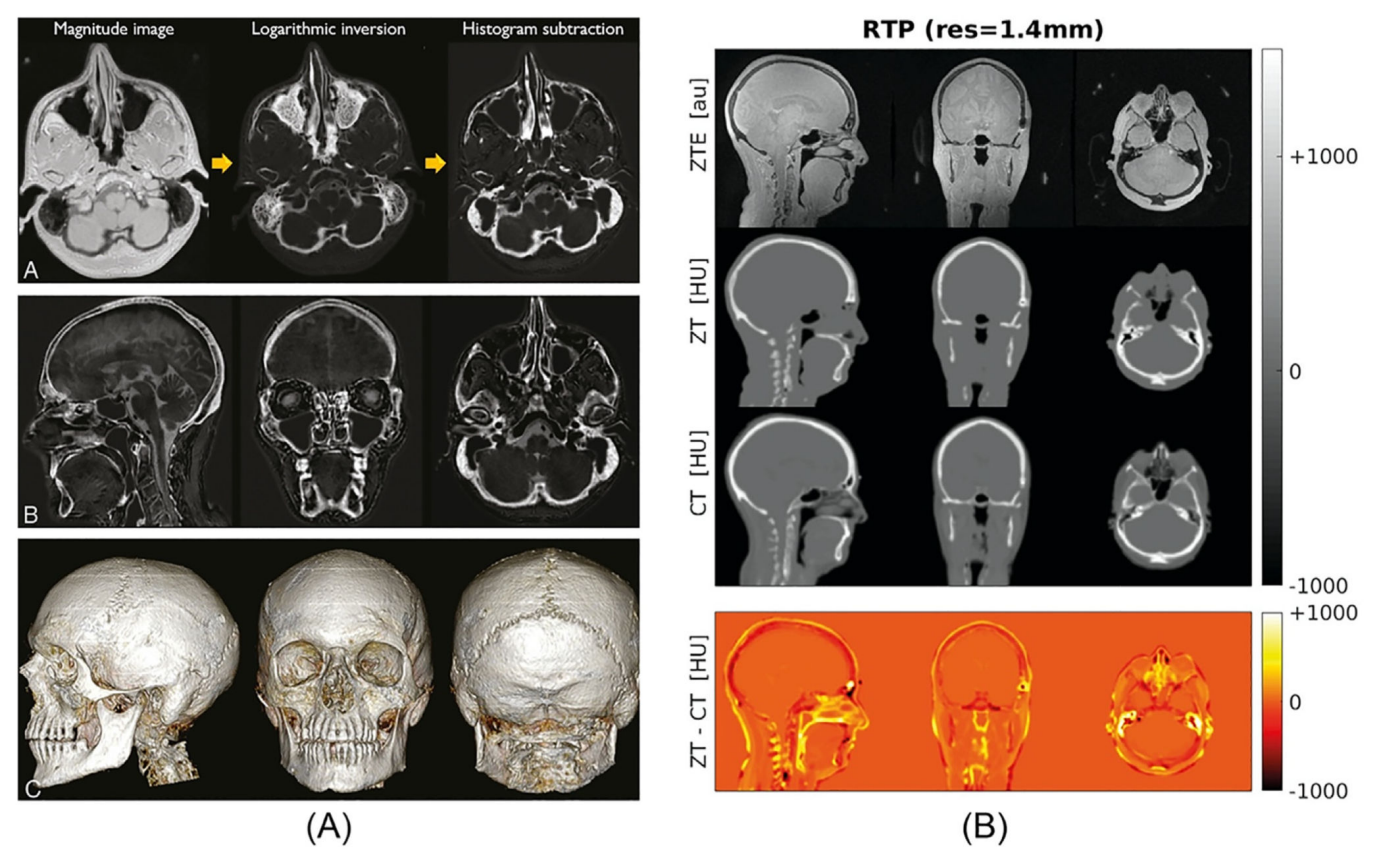
Ultra-short T_2_ PD imaging with ZTE. (**A**) Skull segmentation. Republished with permission of the American Society of Neuroradiology from Ref. [146]; permission conveyed through Copyright Clearance Center, Inc. (**B**) Generation of pseudo CT images from ZTE in Hounsfield units, compared to acquired CT (Abbreviations: **ZT** – ZTE-derived pseudo-CT, **RTP** – Radiation Therapy Planning, **Res** – Resolution, **HU** – Hounsfield Units). Reproduced with permission of John Wiley and Sons from Ref. [147], © 2018 International Society for Magnetic Resonance in Medicine.

The utility of ZTE for bone imaging and segmentation has been demonstrated in several studies [76,144–146], as exemplified in Fig. 15A. In order to contrast bone against surrounding soft-tissue, a low flip angle PD-weighted ZTE acquisition with minimal T_1_ saturation is used. Chemical shift interferences at fat–water interfaces must be avoided by using a pixel bandwidth larger than the fat–water chemical shift (i.e., 3.5 ppm) [79].

ZTE images can also be converted to pseudo computed tomography (CT) images as required for attenuation correction in combined positron emission tomography (PET) and MRI acquisitions, and MR-only radiation therapy (RT) dose planning [147,148] (see Fig. 15B). Inoue et al. also demonstrated that PD weighted ZTE together with MRA can be a useful tool for endoscopic endonasal transsphenoidal surgery planning, as it allows visualisation of internal carotid arteries as well as cortical bone [149].

**Fig. 16:**
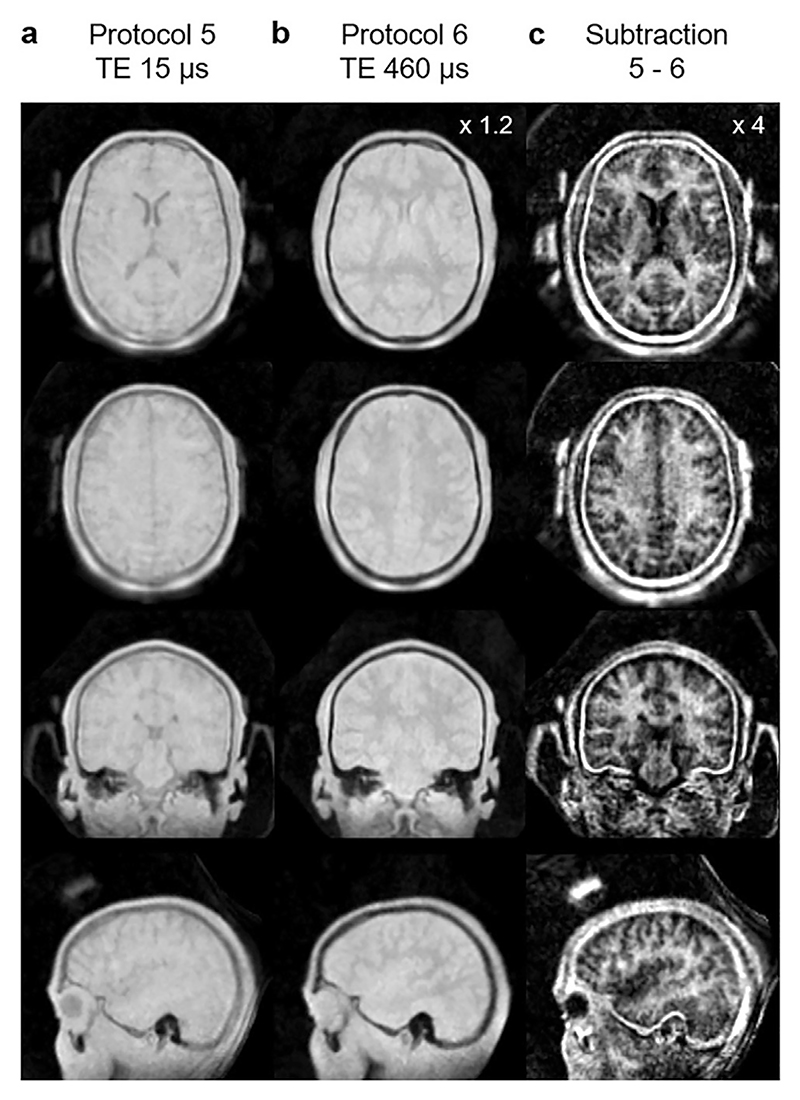
Direct myelin imaging with ZTE by Weiger et al. Subtraction of two images with different effective TE yields a qualitative image with contrast between white and grey matter. Reproduced with permission of Elsevier from Ref. [77] under a CC BY-NC-ND 4.0 license.

Imaging of myelin in the central nervous system is of great interest due to its involvement in numerous diseases, as well as in normal development [150]. Direct imaging of myelin has long been considered impossible due to the ultra-short T_2_ of myelin [151,152]. Methods have therefore been developed to probe different proxies for myelin such as ihMT [128], or measurement of water trapped within the myelin lipid bilayers by multi-component T_2_ mapping [153]. However, Weiger et al. recently demonstrated direct imaging of ultra-short T_2_ components in the brain, attributable in large part to myelin, using ZTE on a customized 3T MR system with a readout bandwidth up to 2000 kHz [77]. By subtracting two images acquired with different bandwidths, resulting in effective TEs of 15 and 460 μs, they produced a qualitative image with high sensitivity to white matter (see Fig. 16). The total acquisition time was ≈45 min. Jang et al. have carried out a similar study of imaging ultra-short T_2_ components in the brain, but using IR prepared ZTE with a unipolar gradient echo on each spoke, meaning that the sequence is no longer silent [154].

### 4.9 Other ZTE applications

In addition to the applications described above, ZTE has also been explored for other applications such as MR electrical property (EP) tomography [155] and MR thermometry [156]. In EP tomography, conductivity and permittivity maps are estimated from magnitude and phase variations of the RF magnetic field governed by Maxwell’s equations [157]. Lee et al. [155] and more recently also Soullié et al. [158] developed an algorithm for EP mapping based on the product of the transmit $\left(B_{1}^{+}\right)$ and receive $\left(B_{1}^{-}\right)$ RF fields [159]. For this purpose, high bandwidth, low-flip angle PD-weighted ZTE images can be used as an approximation of $B_{1}^{-}\cdot B_{1}^{+}$.

The ZTE-VFA method can be extended for rapid measurement of relative temperature changes based on T_1_ temperature dependence [156,160]. In addition to temperature monitoring in soft tissue, ZTE also permits assessing temperature changes in bone structures (e.g., skull) which is important for MR-guided thermal therapies such as high-intensity focused ultrasound (HiFU) [161].

## 5 ZTE for functional neuroimaging

Functional MRI utilises BOLD contrast to study brain function [162]. This contrast can be observed in T_2_- or T_2_*-weighted acquisitions. A standard ZTE acquisition, e.g., RUFIS, has TE = 0, i.e., no T_2_ or T_2_* weighting, and hence does not display BOLD contrast. To achieve functional BOLD contrast with a ZTE sequence, the readout can be preceded by a T_2_-preparation module, which has been demonstrated at both 3T [74] and 7T [111]. In the study at 3T, applications included motor and auditory tasks in four volunteers, where T_2_-prepared RUFIS showed lower sensitivity but improved spatial specificity compared to gradient echo EPI and spin echo EPI. The disadvantage of T_2_-prepared ZTE fMRI is increased acquisition time due to the T_2_ preparation and T_1_-recovery period between segments.

**Fig. 17:**
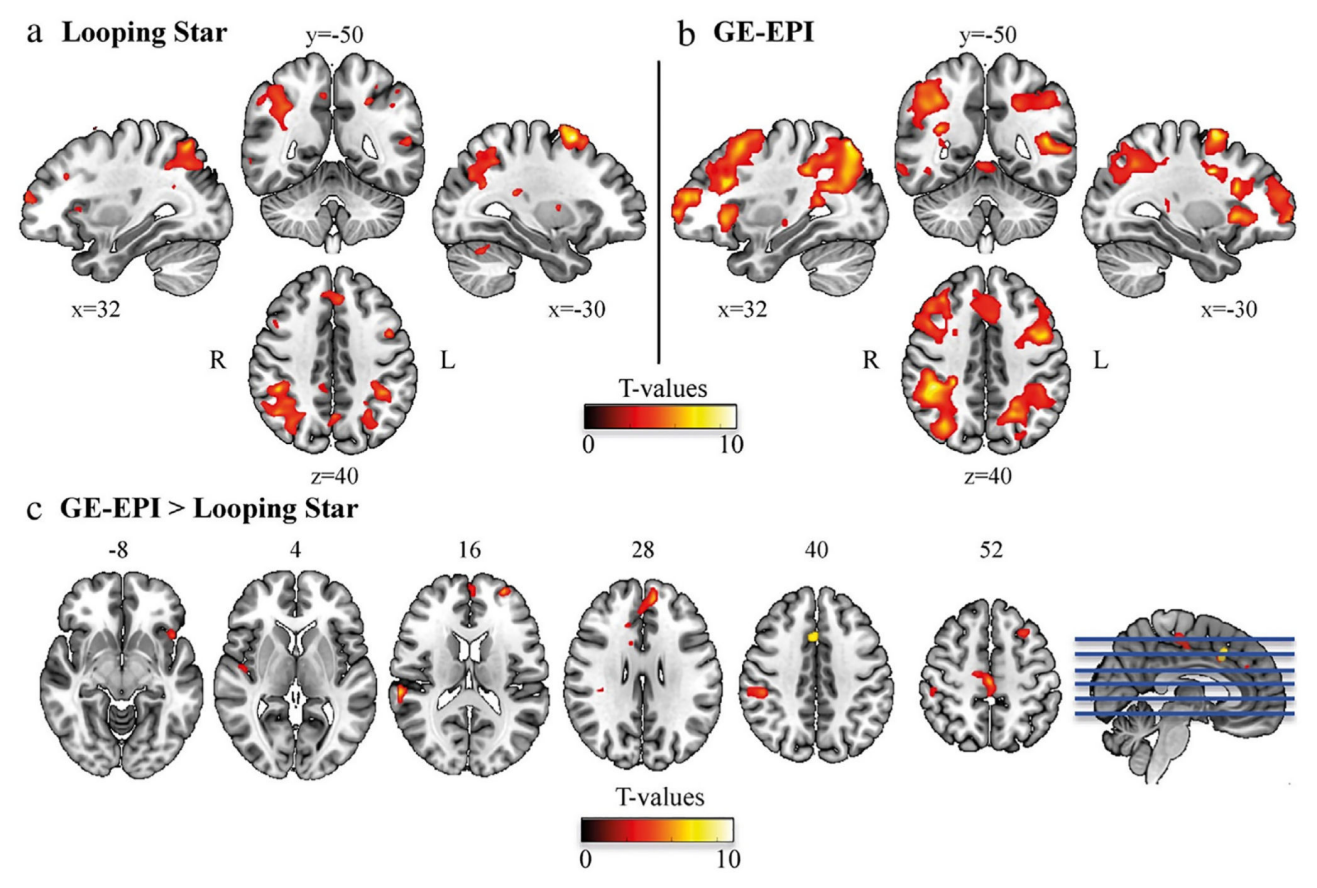
Images from Dionisio-Parra et al. showing second-level results from an N-back working memory task using (**a**) Looping Star and (**b**) Gradient Echo (GE) EPI. (**c**) Results from a paired *t*-test between the two techniques. Reproduced with permission of John Wiley and Sons from Ref. [72], under a CC BY-NC 4.0 license.

The Looping Star sequence can produce T_2_* contrast in the steady state and is therefore a more efficient option for ZTE fMRI. Dionisio-Parra et al. demonstrated the use of Looping Star with a single echo acquisition, as commonly used for GRE-EPI, with visual working memory and resting state paradigms [72] (see Fig. 17). Using a similar acquisition, Wiesinger et al. demonstrated sensitivity to a motor paradigm [71]. Multi-echo fMRI is increasingly being employed due to the benefits of combining echoes, such as reducing the impact of physiological noise. Preliminary results of the multi-echo capability of Looping Star have been presented along- side both block-design and event-related auditory paradigms [73,163], demonstrating good sensitivity to these more complex cognitive tasks.

Mangia et al. have shown preliminary human in vivo results using SWIFT for fMRI [164]. Since SWIFT has TE = 0 it does not produce BOLD contrast. Lehto et al. demonstrated that the main contribution to the observed signal changes in SWIFT fMRI is most likely due to increased blood flow during neuronal activity [165]. Functional imaging with SWIFT has mainly been applied in rodent experiments using either deep brain stimulation (DBS) [165,166] or simultaneous EEG [167]. The advantages of SWIFT compared to standard sequences in these situations are twofold. First, susceptibility artefacts are reduced, resulting in better image quality around the electrodes. Secondly, the low gradient switching rate, which ensures silent acquisition, also results in lower induced currents in the DBS and EEG electrodes and leads. This is particularly important for EEG, where the fast gradient switching can distort the EEG signal [168].

## 6 Discussion

A ZTE pulse sequence is in essence the simplest spatially-encoded MR pulse sequence one could envision. With RF excitation in the presence of the readout gradient, FID readout and minimal gradient switching between excitations, the acquisition produces low acoustic noise. It also enables very short TRs, on the order of 1 ms, and near 100% sampling efficiency. The native contrast in a ZTE sequence is PD or T_1_, making these contrasts particularly well suited for ZTE, including applications such as bone imaging and ZTE-MP2RAGE. Using Looping Star, T_2_* contrast can also be achieved for fMRI and QSM imaging. To obtain additional image contrasts, magnetization preparation techniques must be employed.

Translation of silent ZTE imaging to clinical settings will require the full suite of clinical image contrasts to be available, including PD, T_1_, T_2_ and DW. While PD- and T_1_-weighted images are easily obtained, high quality T_2_-weighted scans exhibit some difficulties with ZTE given its FID acquisition nature. Using multi-parametric ZTE it is possible to perform combined PD, T_1_ and T_2_ mapping, which then can be used for generating contrast weighted images with different T_2_ contrast, as shown in Fig. 9. Synthetic imaging has some limitations though, especially noticeable in T_2_ FLAIR images as highlighted in previous studies [169,170]. Wang et al. demonstrated a method for MR image synthesis using a deep learning network which was able to remove many of the artefacts commonly seen in synthetic T_2_ FLAIR, such as edge enhancement at tissue interfaces [170]. Furthermore, if the end goal is to perform silent T_2_-weighted imaging, spin echo based sequences with smooth gradient waveform should also be considered and included in future evaluations of silent imaging protocols [63,171,172].

The protocol for DW-ZTE by Yuan et al. showed that diffusion contrast can be achieved, but their acquisition suffered from long acquisition times [69]. To enable clinical translation, further research should investigate options for optimal acquisition strategies for combination of the phase cycling and eliminative averaging required for this approach. Again, an alternative to DW-ZTE could be EPI based sequences with sinusoidal gradients [173] and reduced slew rates [174] which have shown promise to reduce the acoustic noise. For both T_2_- and DW-prepared ZTE, advanced reconstruction methods, such as deep learning as shown in Fig. 8, could be a way to improve image quality and reduce acquisition time, making them more competitive compared to their non-silent equivalents.

In the field of neurodegeneration, imaging methods for studying myelin are of great interest. Recent developments of ZTE sequences have demonstrated the capacity for sensitizing images to the myelin bilayer. The approach by Weiger et al. utilizing image subtraction at different TEs is an impressive methodological advancement, but requires long acquisition times [77]. With IR preparation, Jang et al. demonstrated a myelin-sensitized protocol in clinically feasible times, but at the expense of increased acoustic noise from the unipolar gradient echo [154]. Both of these ZTE methods produce images with a signal intensity proportional to the observable MR signal from short-relaxation-time species, which in brain tissue is largely attributable to myelin. Thus, while only semi-quantitative, they represent the closest approaches to date to direct myelin imaging. An alternative approach is ihMT weighted ZTE which is an indirect measure of myelin through magnetization transfer. While giving only an indirect measure of myelin, ihMT has shorter acquisition time, maintains silent operation, and has been shown through histological studies to be highly sensitive to myelin [130].

One patient cohort where silent imaging could find numerous applications is in neonatal imaging, where acoustic noise reduction is required both for hearing protection and to enable scanning during natural sleep [18]. Acoustic noise can be reduced actively using modifications to conventional pulse sequences such as lower slew rate, but also passively using padding inside the scanner in addition to conventional hearing protection [18,175]. In the developing human connectome project (DHCP), a method of ramping up the gradient amplitude to the desired operational level over a number of repetitions of the sequence in the beginning of the acquisition, in their case a period of 5 s, is used to reduce the startle response, thus not disturbing natural sleep [19]. Considering the constraints put on neonatal imaging with regard to acoustic noise, we believe this is a promising area for adoption of silent ZTE neuroimaging techniques.

Silent MRI techniques could also be helpful for *in utero* MRI, where it is not possible to fit the foetus with hearing protection. While several studies have shown that the surrounding maternal tissue is enough to reduce the acoustic noise down to non-harmful levels [176–180], using a truly quiet sequence would minimize any remaining parental anxiety over this issue.

Finally, in the literature, ZTE sequences go by many different names. RUFIS was one of the first silent ZTE sequences with continuous gradients [45], using algebraic reconstruction to recover the dead-time gap, whereas recent implementations of ZTE on clinical scanners typically use WASPI or PETRA. We recommend using the term ZTE for this general category of pulse sequences and, when applicable, specifying which dead-time gap recovery method is used (PETRA, WASPI, HYFI or algebraic reconstruction). This is particularly useful for neuroimaging applications, where the choice of dead-time gap method has less impact on the image quality and characteristics than in imaging of ultra-short T_2_ species [54,143]. Unified terminology will also help accelerate the adoption of these imaging techniques in research studies and clinical practice.

## 7 Conclusions

Silent ZTE sequences show great potential for use in neuroimaging. The most obvious benefit of swapping to such sequences is the large reduction in acoustic noise, which will greatly increase patient comfort, reduce anxiety, improve communication between the radiographer and subject, and enable a wider range of research into auditory conditions. While ZTE sequences are well suited for PD and T_1_ contrasts, and the new Looping Star sequence provides T_2_* and susceptibility contrasts, some standard clinical contrasts such as T_2_ and diffusion remain challenging. However, rapid progress is being made with these, and a truly silent comprehensive protocol looks likely to be feasible in the near future.

## Figures and Tables

**Table 1:** Acoustic noise measurements with silent ZTE sequences from published studies with comparisons to conventional sequences, when available. Values have been rounded to the same precision for comparison, and differences between sequences and ambient noise levels were calculated before rounding.

			Silent				Comparison						
Modality	B_0_	*L_AMB_*	Sequence	*L_ZTE_*	*L_ZTE_-L_AMB_*		Sequence	*L_STD_*	*L_STD_-L_AMB_*	* *	*L_STD_-L_ZTE_*	Unit	Ref
Structural T_1_	3T	69	RUFIS	69	< 1		IR-SPGR	105	36		36	dB	[62]**
Structural T_1_	3T	52	RUFIS	53	< 1		IR-SE	82	30		30	dB	[63]*
Structural T_1_	7T	53	RUFIS	55	2		IR-SPGR	90	37		35	dBA	[64]*
Structural T_1_	3T	48	PETRA	51	3		MPRAGE	78	30		27	dBA	[65]*
Structural T_1_	3T	53	PETRA	58	5		MPRAGE	87	34		29	dBA	[66]*
VFA T_1_	3T	70	RUFIS	70	< 1		SPGR	103	33		33	dBA	[67]**
MRA	3T	55	RUFIS	58	3		TOF	93	38		35	dB	[68]*
DWI	3T	51	DWI-RUFIS	54	3		DWI-EPI	85	34		31	dB	[69]*
T_2_^*^ / QSM	3T	67	ZTE-BURST ^†^	76	9		mGRE	103	37		27	dBA	[70]**
T_2_^*^ / QSM	3T	64	Looping Star ^††^	73	8		N/A	–	–		–	dBA	[71]**
fMRI	3T	64	Looping Star	67	3		N/A	–	–		–	dBA	[71]**
fMRI	3T	71	Looping Star	71	1		GRE-EPI	103	32		32	dBA	[72]**
fMRI	3T	N/A	Looping Star	74	–		GRE-EPI	108	–		34	dBA	[73]**
fMRI	3T	72	T_2_-prep RUFIS	75	2		GRE-EPI	114	42		39	dBA	[74]**

Abbreviations: **B**_**0**_: Main magnetic field strength, **L**_**AMB**_: Ambient Sound Pressure Level (SPL), **L**_**ZTE**_: ZTE SPL, **L**_**STD**_: Non-ZTE comparison sequence SPL, **IR**: Inversion Recovery, **SPGR**: Spoiled Gradient Echo, **SE**: Spin Echo, **MPRAGE**: Magnetization Prepared Rapid Gradient Echo, **VFA**: Variable Flip Angle, **MRA**: Magnetic Resonance Angiography, **TOF**: Time of Flight, **DWI**: Diffusion Weighted Imaging, **QSM**: Quantitative Susceptibility Mapping, **mGRE**: Multi Echo Gradient Echo, **GRE-EPI**: Gradient Echo EPI, **N/A**: Not available.

* Microphone placements: *outside the bore*

** *inside the bore*. Study specific notes:

† 1 mm protocol

†† 0.8 mm protocol.

## Glossary

*3D:*: Three dimensional
*ASL:*: Arterial Spin Labelling
*ASPIR:*: Adiabatic Spectral Inversion Recovery
*B*_0_: Static magnetic field
*B*_1_: Radio-frequency magnetic field
$B_{1}^{+}$: Transmit radio-frequency magnetic field
$B_{1}^{-}$: Receive radio-frequency magnetic field
*BLAST:*: Back-Projection Low Angle Shot
*BOLD:*: Blood-Oxygen Level Dependent
*BRAVO:*: Brain Volume Imaging
*BW:*: Bandwidth
*CT:*: Computed Tomography
*CTA:*: Computed Tomography Angiogram
*dBA:*: A-weighted dB
*DBS:*: Deep Brain Stimulation
*DW:*: Diffusion Weighted
*EEG:*: Electroencephalography
*EP:*: Electrical Property
*EPI:*: Echo Planar Imaging
*FA:*: Flip Angle
*FID:*: Free Induction Decay
*FOV:*: Field of View
*FLASH:*: Fast Low-Angle Shot
*GRE:*: Gradient Echo
*FLAIR:*: Fluid Attenuated Inversion Recovery
*fMRI:*: Functional MRI
*FOV:*: Field of view
*HYFI:*: Hybrid Filling
*ihMT:*: Inhomogeneous Magnetization Transfer
*IR:*: Inversion Recovery
*mBIR4:*: modified B1 Insensitive Rotation Adiabatic RF Pulse with 4 segments
*MP:*: Magnetization Preparation
*MPRAGE:*: Magnetization Prepared Rapid Gradient Echo
*MP2RAGE:*: Magnetization Prepared 2 Rapid Gradient Echoes
*MR:*: Magnetic Resonance
*MRA:*: Magnetic Resonance Angiography
*MRI:*: Magnetic Resonance Imaging
*MT:*: Magnetization Transfer
*PD:*: Proton Density
*PETRA:*: Pointwise Encoding Time reduction with Radial Acquisition
*RF:*: Radio Frequency
*RUFIS:*: Rotating Ultra-Fast Imaging Sequence
*QSM:*: Quantitative Susceptibility Mapping
*RT:*: Radiation Therapy
*SNR:*: Signal to Noise Ratio
*SPGR:*: Spoiled Gradient Echo
*SWIFT:*: Sweep Imaging with Fourier Transform
*T*_1_: Longitudinal relaxation time
*T*_2_: Transverse relaxation time
*T*_2_*: Apparent transverse relaxation time
*TE:*: Echo Time
*TOF:*: Time of flight
*TR:*: Repetition Time
*UTE:*: Ultra-short echo time
*VFA:*: Variable Flip Angle
*WASPI:*: Water and fat-Suppressed proton Projection MRI
*ZTE:*: Zero Echo Time
